# Dietary Transitions and Health Outcomes in Four Populations – Systematic Review

**DOI:** 10.3389/fnut.2022.748305

**Published:** 2022-02-09

**Authors:** Mariel Pressler, Julie Devinsky, Miranda Duster, Joyce H. Lee, Courtney S. Glick, Samson Wiener, Juliana Laze, Daniel Friedman, Timothy Roberts, Orrin Devinsky

**Affiliations:** ^1^Department of Neurology, NYU Grossman School of Medicine, New York, NY, United States; ^2^NYU Health Sciences Library, New York, NY, United States

**Keywords:** obesity, diabetes, western diseases, refined carbohydrates, nutrition transition

## Abstract

**Importance:**

Non-communicable chronic diseases (NCDs) such as obesity, type 2 diabetes, heart disease, and cancer were rare among non-western populations with traditional diets and lifestyles. As populations transitioned toward industrialized diets and lifestyles, NCDs developed.

**Objective:**

We performed a systematic literature review to examine the effects of diet and lifestyle transitions on NCDs.

**Evidence Review:**

We identified 22 populations that underwent a nutrition transition, eleven of which had sufficient data. Of these, we chose four populations with diverse geographies, diets and lifestyles who underwent a dietary and lifestyle transition and explored the relationship between dietary changes and health outcomes. We excluded populations with features overlapping with selected populations or with complicating factors such as inadequate data, subgroups, and different study methodologies over different periods. The selected populations were Yemenite Jews, Tokelauans, Tanushimaru Japanese, and Maasai. We also review transition data from seven excluded populations (Pima, Navajo, Aboriginal Australians, South African Natal Indians and Zulu speakers, Inuit, and Hadza) to assess for bias.

**Findings:**

The three groups that replaced saturated fats (SFA) from animal (Yemenite Jews, Maasai) or plants (Tokelau) with refined carbohydrates had negative health outcomes (e.g., increased obesity, diabetes, heart disease). Yemenites reduced SFA consumption by >40% post-transition but men's BMI increased 19% and diabetes increased ~40-fold. Tokelauans reduced fat, dramatically reduced SFA, and increased sugar intake: obesity and diabetes rose. The Tanushimaruans transitioned to more fats and less carbohydrates and used more anti-hypertensive medications; stroke and breast cancer declined while heart disease was stable. The Maasai transitioned to lower fat, SFA and higher carbohydrates and had increased BMI and diabetes. Similar patterns were observed in the seven other populations.

**Conclusion:**

The nutrient category most strongly associated with negative health outcomes – especially obesity and diabetes – was sugar (increased 600–650% in Yemenite Jews and Tokelauans) and refined carbohydrates (among Maasai, total carbohydrates increased 39% in men and 362% in women), while increased calories was less strongly associated with these disorders. Across 11 populations, NCDs were associated with increased refined carbohydrates more than increased calories, reduced activity or other factors, but cannot be attributed to SFA or total fat consumption.

## Key Points

**Question:** What dietary factors contribute to non-communicable chronic diseases (NCDs) among populations transitioning from their original to westernized diets?

**Findings:** Our systemic literature review examined four populations that transitioned from their original to a more westernized diet and lifestyle. We also reviewed seven additional populations that underwent a similar transition. We identified a strong association between NCDs and increased sugar and refined carbohydrate consumption, and weaker associations with increased total calories with reduced physical activity. Neither fat nor saturated fat intake were associated with risk of developing NCDs in any of the populations.

**Meaning:** Increased consumption of sugar and refined carbohydrates were strongly associated with the development of NCDs in all four populations. Increased calories and decreased physical activity were less strongly correlated although both of these measures are imprecisely defined and not quantified in any of these group. Neither fat nor saturated fat intake were associated with NCD risk in any population.

## Introduction

US obesity prevalence increased 313% from 1960 (13.4%) to 2018 (42%) (1), increasing morbidity and mortality, and costing >$150 billion yearly in US direct health care and lost productivity. As obesity rose worldwide, a parallel but delayed rise in T2D, hypertension, heart disease, stroke, cancer, Alzheimer's disease, and gout usually followed. Debate persists on the roles of energy balance, total fat, SFAs, sugar and refined carbohydrates, total calories or inactivity as primary cause(s) of obesity. Medical orthodoxy holds that positive energy balance (calories consumed exceed those expended) causes obesity, while others argue that sugar/refined carbohydrate-induced metabolic alterations drive obesity and NCDs via metabolic syndrome (2–9). They are not mutually exclusive. Medications (e.g., insulin, antipsychotic, antiseizure), medical and psychiatric disorders, stress, sleep deprivation, and other factors influence weight, confounding attempts to definitively link factors such as caloric or sugar consumption and weight. Further, quantifying caloric consumption and activity levels in populations over time is unreliable. Together, these limitations make it difficult to identify causal relationships.

Nutritional causes of disease have focused on heart disease and obesity and less often, on cancer. Keys' Diet-Heart hypothesis (10) posited that dietary saturated fatty acids (SFAs) increased heart disease risk and was supported by the Seven Countries Study and subsequent research (11, 12). Recent Cochrane (11) and American Heart Association Presidential Advisory reviews (13) of randomized controlled trials (RCTs) conclude that replacing SFAs with polyunsaturated fatty acids (PUFAs) in vegetable oils or unrefined carbohydrates - but not with refined carbohydrates and sugars - lowers low-density lipoproteins (LDL) and reduces the risk of all cardiovascular disease (CVD) events by ~14–30% (11, 13). However, no benefits were seen in non-fatal myocardial infarction, coronary heart disease (CHD) deaths, stroke, cancer diagnoses or deaths, or in overall mortality (11). Other expert groups argue that the evidence fails to support any adverse health effects of eating whole-fat dairy or unprocessed meats (14). Further, large prospective epidemiological population studies of single populations, some with >6-fold differences in SFA consumption, and RCTs found little or no relationship between SFA consumption and weight, cholesterol levels or cardiovascular disease (CVD) risk (15–26). Finally, some studies found increased rates of CVD, cancer, or death when PUFAs were substituted for SFAs or in individuals with low cholesterol levels (27, 28).

In the 1970s and 1980s, health agencies extended the recommendation to reduce dietary fat and SFAs from older men at high-risk for CVD to all individuals over age 2 years. No evidence supported this broader recommendation. The low SFA recommendation was generalized to reduce cancer risk and promoted by the National Cancer Institute (29), National Academy of Sciences (29), American Cancer Society (30), and Surgeon General (31, 32). However, population-based studies correlated low cholesterol with higher cancer rates (30, 32, 33), and clinical trials revealed that cholesterol lowering diets or drugs led to increased cancer rates (34–36). Populations consuming larger amounts of animal fat such as the Inuit and Plains Native Americans had very low rates of cancer (37, 38). Since obesity was associated with increased risk of multiple cancers, fat or SFA were linked to cancer indirectly because fats are energy dense. Yet, only populations consuming high fat diets with abundant sugar or other refined carbohydrates had high rates of cancer and other NCDs. Large prospective studies and RCTs failed to support the link between dietary fat or SFAs and cancer (11, 34, 35), and health agencies no longer support fat/SFA reduction to reduce cancer risk (39).

The dietary causes of obesity, heart disease, and NCDs have been studied with 1) animal models of genetic obesity, brain lesions, and overfeeding of calories or selected macro- or micro-nutrients, and drugs; 2) human volunteer studies of variable duration with overfeeding, underfeeding, increased exercise, different macronutrient diets, and hormonal and lipid changes associated with dietary changes; 3) retrospective and prospective observational studies on diet and health outcomes within and between populations; and 4) randomized controlled trials. However, NCDs may be best understood as evolutionary mismatch disorders. Evolutionary mismatch refers to advantageous traits that evolved in one environment, but became maladaptive after a rapid environmental change (40). NCDs are rare or absent among hunter-gatherer, nomadic, foraging-gardening, and agricultural-dependent populations with diverse genetics, diets and lifestyles (40). Some groups consumed primarily plant-based carbohydrates, others primarily animal fat and protein. NCDs were rare in all. These diets were minimally processed, lacking refined sugar, white flour, white rice, vegetable oils, chemical preservatives or meat from animals bred for high fat content or fed corn. When non-western populations transition to western diets and lifestyles, NCDs follow (41–47). Americans consume a typical western diet: high total calories and mostly processed foods including sugars, white flour and rice, meats (chicken, pork, beef), and dairy; with limited vegetables and fruit (48–50). Our domesticated plants and animals were bred for increased sugar [e.g., grapes (51) and apples (52)] and intramuscular fat (e.g., Aberdeen angus cow) while decreasing fiber (e.g., fruits). Westernization reduced physical activity as machines replaced manual labor and self-powered transport; while for others, a fitness revolution increased physical activity (41). In contrast to RCTs, transitioning populations offer the opportunity to observe natural changes in population health that occur with an increase in western diet and lifestyle over extended periods.

Most cultures have moved toward a more industrialized western diet, with more sugar, refined flour and rice, and processed oils and meats. Greater availability of refined carbohydrates reflected lower cost, longer shelf-life, ready-to-eat, and possibly more addictive; fueling increased consumption that drove poor health outcomes (42–44). Although this western transition occurred in hundreds of populations, few studies systematically quantified changes in diet, physical activity, stress and other risk factors with health outcomes. Two longitudinal studies included in our analysis are the Tokelau Island Migrant Study and the Seven Countries Study, but both had limited data on diet and health outcomes and did not prospectively assess changes in total calories, unrefined vs. refined carbohydrates, physical activity, medication use, and many other factors.

Studying populations transitioning toward western diets may inform pathogenic factors underlying some NCDs. What dietary or lifestyle changes are critical - total calories, reduced physical activity, sugar and refined carbohydrates, processed foods, mental stress, reduced sleep, medications, or other factors? Given the many limitations, even for rigorous prospective studies, we systematically reviewed the health outcomes of four extensively studied, diverse populations before and after a nutrition transition. We also reviewed data from seven additional populations to assess potential bias in our selection. Our goal was to identify common factors associated with NCDs in these populations.

## Methods

### Selection of Populations

We systematically reviewed transitional populations to examine dietary effects on NCD risk. A review protocol was registered with PROSPERO, accessible at (https://www.crd.york.ac.uk/prospero/display_record.php?RecordID=164532). We first surveyed the literature on populations that had undergone a nutrition transition. We searched combinations of “nutrition, diet, transition, non-western, hunter-gatherer and Indigenous” on PubMed and identified over 15,000 articles, whose titles and when relevant, abstracts were reviewed. We also reviewed books on this topic and their references (33, 45–47, 51, 53–62).

We identified 22 groups with sufficient data to warrant a more detailed review: Arawak, Aboriginal Australians, Chinese, Ethiopians, Indians, Inuit, Mapuche, Maasai, Mongols, Natal Indians in South Africa, Navajo, Nepalese, Pima, South Koreans, Tokelauns (New Zealand), Tanushimaru (Japan), Tsimane, West Africans, Hadza, Yemenite Jews, Yukon, and South African Zulu speakers. We initially reviewed these populations and rated the data quality on dietary changes pre- to post-transition. For each group, original research studies and reviews were assessed to determine the adequacy of data on pre- and post-transition diets and health outcomes. These included a minimum of pre- and post- transition dietary macronutrient composition and at least one health outcome (e.g., changes in weight, diabetes, or hypertension prevalence). Eleven were identified: Tanushimaru, Pima, Navajo, Hadza, Inuit, Tokelauns, Maasai, Aboriginal Australians, South African Natal Indians and Zulu speakers, and Yemenite Jews. After re-reviewing dietary transition data, we selected four populations that met our criteria: Yemenite Jews (Yemen/Israel), Tokelauans (New Zealand), Tanushimaru (Japan), and Maasai (Kenya/Tanzania). Given the number of papers and overlapping features, we excluded seven due to limitations of data or overlap (see below). We systematically reviewed the four with diverse locations and pre-transition diets (Yemen-Israel; Tokelau in South Pacific; Tanushimaru, Japan; Maasai in East Africa).

The Pima and Navajo were excluded from systematic review since their transition began in the mid-19^th^ century and data from that period were limited. The Inuit and Aboriginal Australians were excluded since there were multiple populations studied over long periods and distances. The data on pre-transition diet was insufficient and relied largely on anecdotal evidence. Similar but better documented dietary changes occurred in the Maasai (initial high fat diet, similar to Inuit) and Tanushimaru (initial high carbohydrate diet, similar to Rural Chinese) over more limited times and distances. The Hadza were excluded since limited data directly compared pre and post transition diets. The South African Natal Indians and Zulu speakers were excluded due to limited data and their major dietary change was more sugar and refined carbohydrates, similar to Yemenite Jews, Tokelauans and Maasai. However, to assess possible bias in selection, we reviewed data from seven excluded groups (Pima, Navajo, Aboriginal Australians, South African Natal Indians and Zulu speakers, Inuit, and Hadza).

### Search Strategy

The review was conducted following the PRISMA Protocol for Systematic Reviews (PRISMA). Co-author (TR), a Medical Librarian, searched for articles on nutrition and disease in the respective populations across: Medline, Embase, PsycInfo and Global Health (searched on the Ovid Platform), NLM's IndexCat, Web of Science and Scopus. Each population group was searched without restriction on date or language. The Ovid Medline Search for the Yemenite Population was included as Supplementary Material.

### Study Selection

The online tool “Covidence” (www.covidence.org) allowed reviewers to independently vote on studies based on inclusion/exclusion criteria. During the first selection round, reviewers voted on the study's title and abstract. For conflicts, two reviewers jointly decided whether to include the study, full texts were obtained, and the reviewers decided if the study was relevant based on the same criteria. Studies were excluded if no full text was available.

### Eligibility Criteria

Studies were included if they had quantitative data on macronutrients before or after transitioning from their traditional to a more western diet/lifestyle and evaluated >1 health outcome. All studies that met these criteria were included. We excluded studies if they only assessed a certain sub-population, were opinion pieces, or evaluated an intervention.

### Group Characteristics/Transition Definition

Of the four systematically reviewed groups, three underwent a geographic migration (Yemenites, Tokelauans, and Maasai) while the Tanushimaru remained in their village. For each group, comparative data were limited to adults and for the Maasai and Tanushimaru, to men. Genetic changes were unlikely to contribute to differences in any populations given the short interval before and after transition. Yemenite Jews emigrated from Israel to Yemen ~2,000 years ago and lived apart from Ashkenazi and Sephardic Jews, with genetic, diet and lifestyle differences. There were three emigrations back to Israel: (1) ~5,000 between 1881–1914; (2) ~16,000 during the British Mandate 1917–1947, and (3) ~50,000 after Israeli statehood in 1949–1950 (63). For Yemenite Israelis, pre-transition included their time in Yemen or living in Israel for <10 years in 1960; post-transition included the first two emigrations who lived in Israel for >20 years in 1960. Data on their diet in Yemen was obtained from interviewing immigrants in Israel.

Tokelau is a New Zealand protectorate comprised of three remote South Pacific atolls. Overcrowding led to the Tokelau Resettlement Program in 1966, with ~1000 Tokelauans migrating to New Zealand over six years while western food availability increased in Tokelau (64). The pre-transition group lived in Tokelau before 1966 or during the first Tokelau Immigrant Migration Study (TIMS) survey in 1968–1971; the post-transition group lived in New Zealand at the third survey in 1982. Migrants to New Zealand averaged 8 years younger than non-migrants who remained.

The Seven Countries Study began in 1957 as the first prospective longitudinal study of dietary factors associated with cardiovascular disease across international groups, mainly rural populations. Tanushimaru, a farming village, was one of two Japanese male cohorts aged 40–59 years. Tanushimaruans experienced changes in diet and lifestyle after 1958, when governmental initiatives including dietary and economic changes were enacted (65). Pre-transition diet was before 1958 and post-transition was after.

The Maasai are pastoralists in Tanzania and Kenya, with 15 subtribes. The mens' traditional diet is blood, milk (from Zebu cattle) and meat (primarily sheep and goats). In the 1960s-70s, many transitioned to government-supported ranching programs, with their original diet progressively replaced by maize, grains and cereals, or moved to cities with more western diets. The main transition occurred in 1950s when the British reduced the cattle grazing area, forcing lifestyle and dietary changes. (66). Pre-transition was the traditional diet and rural lifestyle, post-transition was after moving to towns with more store-bought foods.

### Data Analysis

Quantitative and qualitative data are summarized in Supplementary Table 1, including study populations, methods, macronutrient data, and health outcomes for each population before and after transition. Studies were grouped by population. All data was reviewed by two nutritionist-dieticians (CSG, JD) who subcategorized macronutrient data based on food composition.

### Nutrient Calculations

We examined diet outcomes, such as total calories, percent of different macronutrients (carbohydrates, proteins, fats), subtypes of carbohydrates and types of fats, and health outcomes (e.g., BMI, diabetes, hypertension, total serum cholesterol). Individual studies reported nutrient data in different ways (e.g., grams of macronutrients, % macronutrients or types of food consumed). We extrapolated dietary composition based on published data from multiple studies and pooled them, reporting nutrient intakes as ranges of percentages in Table 1. For percentages of specific carbohydrate and fatty acid types, we used the USDA nutrient database, in combination with expert knowledge from licensed dietitians, to extrapolate food nutrient composition (67).

**Table 1:** Macronutrient and health outcome percent change after transition (systematically reviewed groups).

**Groups**		**Macronutrients (% total kcal)**			**Types of fat (% total fat)**			**Types of carbohydrates (% total carbs)**			**Health measurements post transition**			
	***Calories (kcal)***	***Fat***	***Protein***	***Carb*.**	***SFA***	***PUFA***	***MUFA***	***Starch***	***Fiber***	***Added sugar***	***BMI***	***Diabetes***	***Hypertension***	***Serum Cholesterol (total)***
Yemenite	↑	↓	↓	↑	↓↓	↑*↑↑↑↑*	↑*↑↑*	↓↓	↓↓	↑*↑↑↑*	↑19%^1^	↑4,733%^1, 2, 3^	SBP: ↑7%^4^ DBP:↑3% (M)^4^	↑7%^4^
Tokelau	↑↑	↓↓	↑↑	↑↑	↓↓	↑*↑↑↑↑*	↑*↑↑*	↑*↑↑*	↓*↓↓*	↑*↑↑↑*	↑19% (M)^5^ ↑14% (F)^5^	↑133% (M)^6^ ↑56% (F)^6^	SBP: ↑7% (M)^5^ ↑8% (F)^5^ DBP: ↑11% (M)^5^ ↑4% (F)^5^	↑9%(M)^7^ ↓1% (F)^7^
Tanushimaru	↓	↑*↑↑*	↑↑	↓↓	-	-	-	-	-	-	↑12% (M)^8^	-	SBP: 0% (M)^8^ DBP: ↑15% (M)^8^	↑24% (M)^8^
Maasai	M: ↑↑ F: ↑↑	M: ↓↓ F: ↓*↓↓*	M: ↓↓ F: ↓*↓↓*	M: ↑↑ F: ↑*↑↑↑*	-	-	-	-	-	-	↑6–11% (M)^9, 10^	↑127% (M)^11^ ↑233% (F)^11^	SBP: ↑7% (M)^9^ DBP: ↑4% (M)^9^	↑27% (M)^12^

*Key: ↑ (0–10%) ↑↑ (11–50%) ↑↑↑ (51–100%) ↑↑↑↑ (100+%). 1. Cohen (68). 2. Cohen (69). 3. Cohen (70). 4. Brunner et al. (71). 5. Salmond et al. (65). 6. Ostbye et al. (72). 7. Stanhope et al. (63). 8. Adachi et al. (73). 9. Day et al. (74). 10. Christensen et al. (75). 11. Christensen et al. (76). 12. Day et al. (74)*.

## Results

### Search Results

After removing duplicates, 2,130 articles were found across the four populations. Titles and abstracts were independently screened by seven reviewers (OD, MP, CSG, JD, SW, MD, JL). We selected studies with dietary data of a population transition from non-western to western-like including at least one health measure or outcome (e.g., weight/BMI, heart disease, cancer, blood pressure).

**Figure 1:**
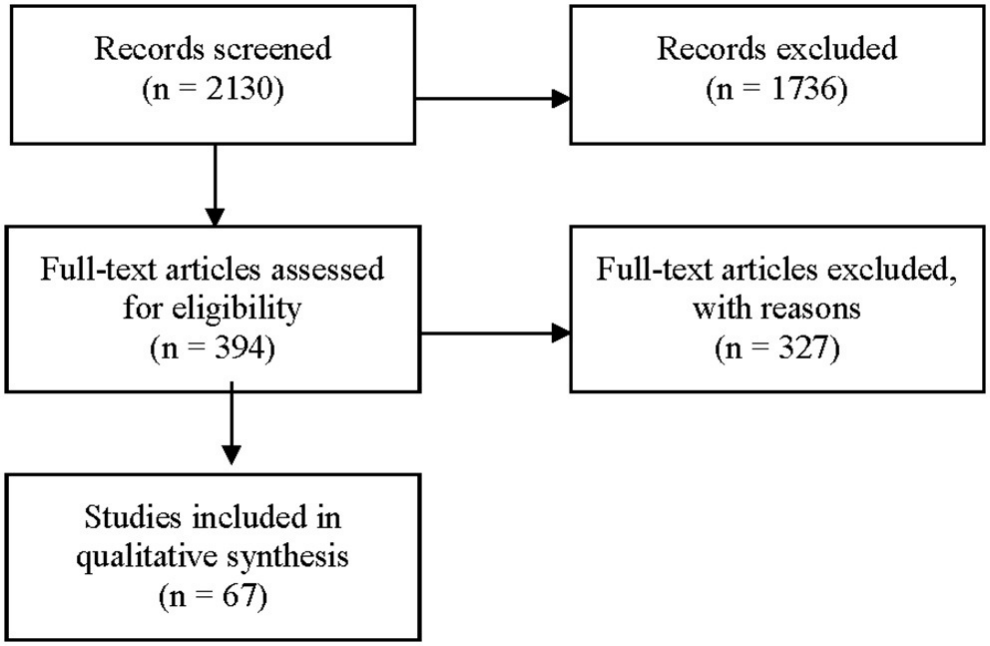
PRISMA flow chart - selecting process. Ovid Medline Search Strategy for the Yemenite Population. August 2019. 1. exp “diet, food, and nutrition”/ or exp “nutritional and metabolic diseases”/ or (diabet* or coronar* or heart* or metabol* or diet* or nutri*).mp. or Body Weight/ or Obesity/ or Overweight/ or Body Image/ or Feeding Behavior/ or Weight Reduction Programs/ or Diet, Reducing/. 2. (Yemen* adj3 Jew*).ti,ab. or “Ethnic Groups”/ and “Israel”/ or Yemen/.

**Figure 2:**
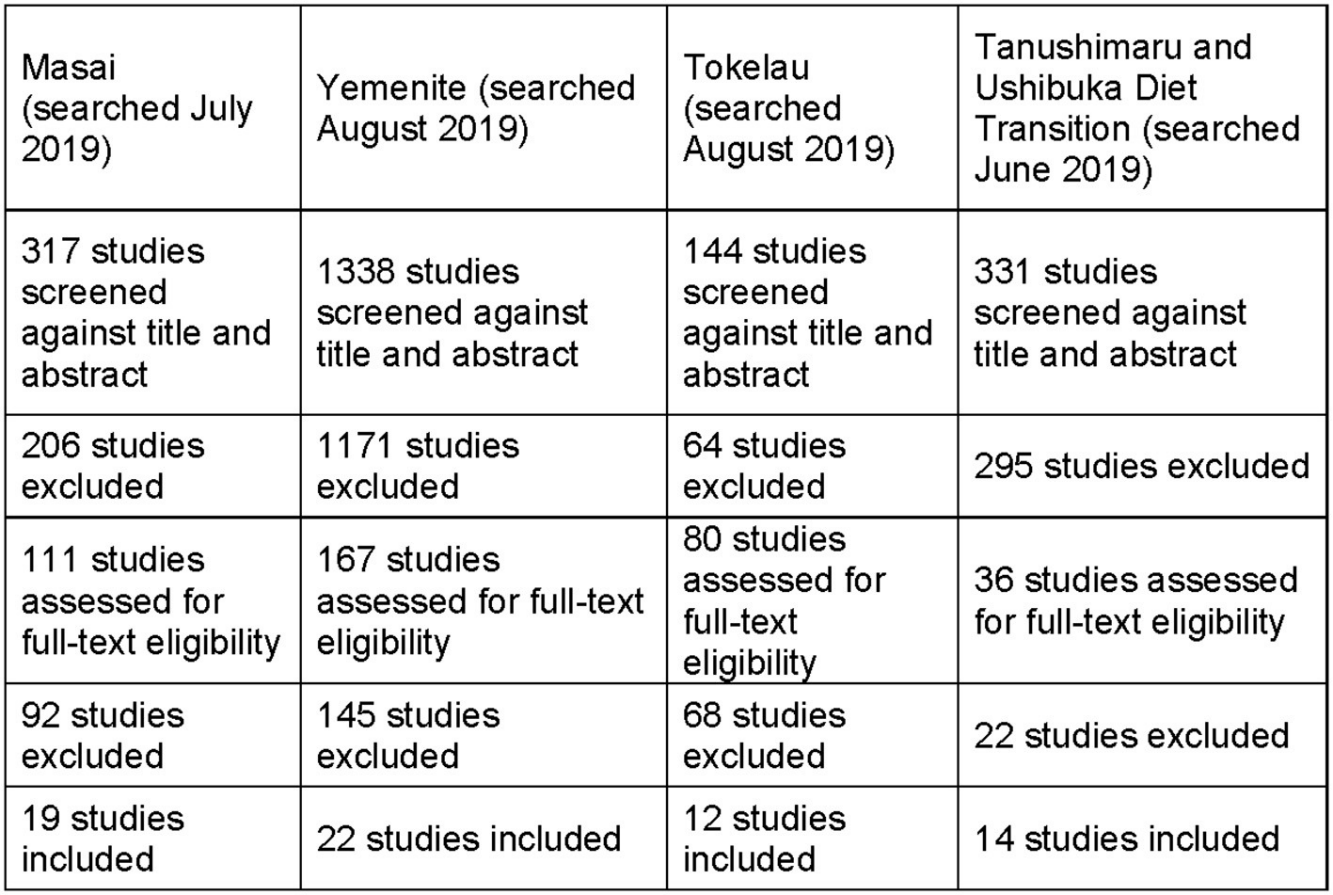
Screening results by population group.

Figure 1 is a PRISMA flow chart showing the study selecting process: 2,130 articles were reviewed by 7 independent reviewers. The first round excluded 1,736 articles. The full texts of the remaining 394 articles were reviewed and 67 met inclusion criteria and their data was extracted (Supplementary Tables 1–5, Figure 2) (64). Primary sources included the TIMS (77) and an Adachi 2020 article on Tanushimaru identified after our original search (73). Secondary sources such as book chapters were identified from bibliographies and were included when original data was identified. Our final selection included 35 observational studies, 12 cross sectional studies, 12 prospective cohort studies, 4 systematic reviews, 2 longitudinal studies, 1 editorial, 1 multivariate analysis, and 1 book.

Pima, Navajo, Aboriginal Australians, South African Natal Indians and Zulu speakers, Inuit, and Hadza, were all independently searched (MP, MD, OD). Key articles with data on pre- and post-transition nutrition and health outcomes were reviewed but were not systematically reviewed.

### Yemenite Jews

#### Diet

Their diet was predominantly carbohydrates before and after migration to Israel; the main post-transition changes were (1) more sugar and less unrefined carbohydrates and (2) more vegetable oils and less animal fats. Post-transition macronutrient were estimated as 10% more calories, 2% less fat energy, 4% less protein energy, and 2% more carbohydrate energy. SFAs decreased 43% while PUFAs increased 240% and monosaturated fatty acids (MUFAs) increased 63%. Starches decreased 12%, fiber decreased 35%, and added sugar increased 600% (55). Compared to Israelis from Europe or America, Yemenites in Israel consumed more calories (19%), bread/cereal (31%), alcohol (230%) and seeds/nuts (348%), but less vegetables (24%), chicken (13%) and dairy (12%) (78).

#### Activity and Lifestyle

Pre-transition, men were active in farming or manual labor. In Israel, men were more sedentary with urban jobs (e.g., driving, office work). There was insufficient data on Yemenite women's activities in Yemen or Israel. In Israel, alcohol comprised 2.5% of adult Yemenites' total calories compared to 1.3% of Israelis from Europe or America. Among Yemenite men, 30% reported smoking >10 cigarettes/day vs. 36% of other Israeli men (48).

#### Health Outcomes

Average BMI increased from 21–23.6 pre-transition to 25–26 post-transition (49). Diabetes prevalence rose from 0.12% in males (M) and 0% in females (F) pre-transition to 0.25% (M) and 0.24% (F) after 20 years in Israel; 2.7% (M) and 3.2% (F) after 30 years, and 6.1% (M) and 3.1% (F) after 40 years (49, 68–70).

Among Yemenites over age 55 years living in Israel for >40 years, 26.3% had diabetes (49). Blood pressure increased: systolic from 116 mmHg to 124 mmHg and diastolic from 74 mmHg to 76 mmHg (71). Serum cholesterol increased from 195 mg/dL to 208 mg/dL.

### Tokelau

#### Diet

The traditional diet was mainly coconuts and fish, with very high total fat (53%) of which 85% was SFAs (77, 79). The prospective TIMS followed most Tokelauns over >18 years (74). The New Zealand migrant diet was 15–20% higher in calories; meat and dairy increased from 1 to 32% of calories. Fat consumption declined 13% and SFA declined 23%; carbohydrates increased 25%, protein increased 25%, MUFAs increased 320% and PUFAs increased 33% (64). Sugar consumption increased in Tokelau and New Zealand; far more for migrants (650; 2 to 13% calories) than non-migrants (400, 2 to 8%) (64, 80).

#### Activity and Lifestyle

Lifestyles differed by gender. Men fished with oared canoes, gathering coconuts, and did work in village but activity declined post-transition on Tokelau as 8–12 h oar-powered canoe trips were reduced to 2–3 h on motorized dinghies (65, 77). As imported foods increased, the work to harvest coconuts, remove their shell and process the copra fruit declined. Migrant men in New Zealand were in active in railway casting shops, forest services and other jobs (77, 81). In Tokelau, women had relatively sedentary lifestyles before and after the transition, as homemakers who prepared and wove plant fibers into mats while sitting and socializing (65). The reported level of activity may underestimate women's activity. In New Zealand, women worked in electrical assembly, clothing factories and cleaning offices, often walking long distances to and from work (77). Automobiles and public transportation in New Zealand partially offset increased activities.

Post-transition 21% of Tokelau men regularly drank alcohol while few women did. In New Zealand (1968–1971), average alcohol consumption increased from 4.3 days/month to 6.7 days/month (63). By 1982, 73% of men and 29% of women Tokelauans in New Zealand regularly drank alcohol (64). In Tokelau (1977), 62% of men and 26% of women smoked cigarettes; in New Zealand (1982), 63% of men and 36% of women smoked (77).

#### Health Outcomes

In 1928, before western foods reached Tokelau, the population had “low levels of coronary heart disease, obesity and diabetes” (64). Blood pressures were low and showed minimal elevation with age. In 1968, the group that migrated to New Zealand was younger (men averaged 11 years and women averaged 7 years) than non-migrants. Ten to 14 years after migration, this younger cohort's BMI increased 19% in men and 14% in women, while the older cohort in Tokelau had BMI increases of 5% in men and women (82). Diabetes prevalence rose in all Tokelau cohorts from 1968 to 1982; in Tokelau from 3.2 to 6.9% in men and from 8.7 to 14.3% in women, in New Zealand from 7.5 to 10.8% in men and 11.7 to 19.9% in women (77).

Cholesterol levels declined slightly from 1968 to 1982 for all Tokelauns for both sexes in Tokelau and New Zealand. In 1968/71, the older non-migrant cohort had slightly higher cholesterol levels than migrants (men 215 vs. 205; women 222 vs. 201) and 1982 (men 191 vs. 185; women 205 vs. 189), attributed to age and higher coconut and SFA consumption in Tokelau. Triglyceride levels rose but remained >30% higher in the younger migrants in 1968/71 (men, 49.5 vs. 27.4) and 1982 (men, 54.5 vs. 41.8). A similar trend was seen in women. The younger migrant cohort had higher LDL and lower HDL than the older migrants after 14 years; more in men than women (64). Over the 14 year study, blood pressures was relatively stable; for older subjects in Tokelau, from 123/71 to 128/74 (men) and 128/75 to 132/77 (women), and for younger migrants, from 121/70 to 129/78 (men) and 120/74 to 129/77(women) (65).

Heart disease was more common in the younger migrants despite their lower cholesterol and smoking rates than in the non-migrants in Tokelau. Electrocardiographic (ECG) evidence of myocardial infarction was found in 1% of migrant men but in no non-migrants. Milder ECG abnormalities were found in 15.6% of migrant men and 8.7% of the older men on Tokelau (77).

### Tanushimaru

#### Diet

The pre-transition diet was very high in unrefined carbohydrates and very low in fat and sugar. This village had one of the lowest CHD death rates of the 16 Seven Countries Study cohorts initially and the lowest after 25 years (66). Post-transition, calories decreased 2%, fat increased 119%, protein increased 31%, and carbohydrates decreased 19%. There was no data on types of fat, starch, fiber, or added sugars. Sugar consumption was very low pre-transition and 45 years post-transition (66, 83).

#### Activity and Lifestyle

Pre-transition, men worked hard (54%) or moderately hard (42%) in agricultural jobs. Post-transition, physical labor decreased (73). Pre-transition, 53% of men reported daily alcohol intake and 56.7% of the men smoked; post-transition, 71% of men consumed alcohol and 16.7% of men smoked (84). There was insufficient data on physical activity, smoking or alcohol consumption for women.

#### Health Outcomes

From 1958 to 2018, men's BMIs increased from 21.7 to 24.4 (73) and serum cholesterol increased from 168 mg/dL to 209 mg/dL. Systolic blood pressure was unchanged at ~133 mmHg post-transition, while diastolic blood pressure increased from 74 pre- to 85 mmHg post-transition (73). Mortality rates from cancer and stroke declined post-transition and mortality rates of myocardial infarction were stable despite a ~27% rise in serum cholesterol. The percent of men on hypertensive medications increased from 3 to 40% from 1958 to 2018 (73).

### Maasai

#### Diet

The traditional Maasai male diet was high in SFA (milk and red meat) and low in carbohydrates with low heart disease rates (85). Post-transition, they consumed less fat and more carbohydrates. Macronutrient consumption differed between men and women pre- (1960) and post- (1980) transition. Post-transition, men's calories increased 19%, fats decreased 14%, proteins decreased 48%, and carbohydrates increased 39%; women's calories increased 14%, fat decreased 62%, protein decreased 73%, and carbohydrates increased 362%. There was insufficient data to calculate percent change for different fats or carbohydrates consumed. Pre-transition SFA intake was >60% of total fats, with total fat comprised >80% of the men's diets. Refined carbohydrates were rarely consumed (76, 85–87).

#### Activity and Lifestyle

Pre-transition, Maasai were very physically active. Post-transition, their lifestyle was more sedentary due to a reduced livestock-to-human ratio, increased land restrictions, and introduction to markets and consumerism (87). Among men, smoking and alcohol use were rare before the transition; post-transition, 11–16% smoked and 15% drank alcohol. The post-transition Maasai ate a primarily traditional diet but government programs led to reductions in fat and SFA and increased carbohydrates and refined carbohydrates (88, 89).

#### Health Outcomes

Pre-transition Maasai had very low rates of heart disease on clinical, EKG and postmortem studies (85, 86). Cholesterol levels were low in rural Maasai in 1964 (mean 125 mg/100ml) (85) and 1971 (135 mg/100ml) (86). Average male BMI increased after the transition from 21.0 in rural to 23.3 in urban settings (only 7 urban men) (90). For women, rural BMI averaged 22.3; few urban Maasai women were studied. One study found mens' average BMI was slightly higher in urban (20.2) than rural settings (18.9) despite the urban group averaging 19 years younger. Rural blood pressures averaged 121/79 for adults in 1928 and were similar (90) in 2017 (116/73 young adult women; 124/77 young adult men) (89, 91). Blood pressures were similar in rural and urban groups, but the rural group averaged 19 years older, suggesting more favorable levels for age. Compared to rural Maasai, younger urbanites had 27% higher cholesterol levels (203 vs. 160 mg/dL) and 10% higher triglyceride levels (159 vs. 144 mg/dl) (74).

Waist circumference was 80.3 cm for rural and 85.3 cm for urban men (76, 92). In 1931, rural Maasai had no evidence of diabetes (91). Among rural Maasai in 2006, 5.2% of men and 7.8% of women had impaired glucose tolerance or diabetes. Among urban Maasai and individuals from other tribes, 11.8% of males and 26% of females had impaired glucose tolerance or diabetes (76). In 2015, diabetes prevalence in adult (>25 y/o) Maasai was 1.1% for men and 0.8% for women; impaired fasting tolerance at 2.4%; diabetes prevalence among all Tanzanians was 8.0% (80).

### Selected Excluded Populations

#### Arizona Pima

The Pima are a southwest Native American group with the highest obesity and diabetes rates in the US in the 1990s. The traditional Pima diet was minimally processed, including wheat, maize, beans, saguaro fruit, diverse mammals, fish, birds, and worms (82, 93). As western settlers diverted water supplies, they relied on government rations, mainly sugar, white flour, lard, as well as store-bought food. Obesity rose before diabetes. In the early 1900s, some older Pima were obese but no diabetes was identified in two surveys (93). By 1971, most adult Pima were obese (94). In 1976, among adults over age 40 years, males averaged 122% above ideal weight, women 149%. In 1994, average adult BMI for was 35.5 for women and 30.8 for men (95, 96). Adult diabetes prevalence was <1% in 1940 (97), 4% in 1955 (98), 30% in 1963 (94), and 38% in 2006 (99). The greatest rise in diabetes occurred in the late 1950s and early 1960s, when caloric consumption was higher than most Americans (2,780 calories/day), but fat consumption was lower (24%; 61% carbohydrate, 15% protein). Compared to genetically related Pima in Mexico, those in Arizona had a 5.5-fold higher rate of diabetes and 37% higher BMI (99, 100). Mexican relatives consumed a less processed diet and were more physically active than those in Arizona.

In 1973, the medical epidemiologist Peter Bennett argued that the Pima's diabetes epidemic resulted from high sugar or caloric consumption; he was uncertain if it was “sugar specifically or … calories in general, which in fact turns out to be really excessive amounts of carbohydrates (101).” The rise of obesity and diabetes in the Arizona Pima likely reflect some combination of excess calories, refined carbohydrates and decreased activity.

#### Navajo

The Navajo diet changed in three stages: (1) contact with the Spanish leading mutton from sheep or goat as their main food (all parts were consumed), (2) after their defeat, forced march and imprisonment by the US Army in 1864-68, and (3) introduction of federal surplus foods and progressive increase in ultra-processed foods. In 1956, the average Navajo BMI was 23.5 for men and for 22.9 women, when their saturated fat intake (mainly mutton and lard-fried bread) was as high or higher than White Americans (102). In 2020, average BMI for Navajo women was 31.2 (103).

In 1947, diabetes was diagnosed in 5/25,000 admissions to a hospital in a relatively young population. In 1968, diabetes prevalence among hospitalized Navajo was 0.6%, and the disease was typically mild (88). By the 1990s, diabetes prevalence was 2–4 fold higher among Navajo than non-Hispanic Whites (90, 92). The major changes to the Navajo diet were increased calories, mainly ultra-processed foods with sugar, white flour, and meat. Compared to the general population, total calories were higher with similar macronutrient percentages. Less than one-third of fat was saturated (104, 105).

#### Aboriginal Australians

Before western contact, Aboriginal Australians were slim with little or no hypertension, diabetes or cardiovascular disease (106, 107). Traditional diets included diverse seeds, tubers, roots, fruits, gums, insects, marsupials, reptiles, birds, fish, mollusks and crustaceans. It was rich in unrefined carbohydrates, fiber, protein, with modest saturated and monounsaturated fat (108, 109). There were no added sugars, refined carbohydrates or other processed foods (108, 109). Those who transitioned to urban settings or remained in home territories and consumed more processed foods developed high rates of obesity, diabetes and cardiovascular disease. Their diet was rich in “flour, sugar, rice, carbonated drinks, alcoholic beverages (beer and port), powdered milk, cheap fatty meat, potatoes, onions, and…(some)…fresh fruit and vegetables (110, 111).” When a group of diabetic subjects returned to their traditional diet, despite 64% of the energy derived from animal sources, their diet was low in fat (13%) and calories (1200/day), their metabolic abnormalities improved or resolved (110). There was limited data on total calories associated with their lifestyle transition, but physical activity declined (112).

#### Natal Indian and Zulu Speaking South Africans

The main Indian migration to South Africa's Natal region occurred in the late 1800s, as indentured workers on sugar, tea and acacia plantations. In the 1950s, they also worked as laborers in fields and mines, but high rates of obesity and diabetes developed. Their diet was rich in “highly refined sugar, foods containing highly refined flour, and polished rice.” These Indian South Africans experienced a “veritable explosion of diabetes”; “one in three middle-aged men were diabetic.” Many consumed low-calorie diets (e.g., 1600–2000 daily) despite physically active work. Per capita sugar consumption was 80 pounds vs. 12 pounds in India. In Natal, diabetes prevalence was 30-fold higher than in India (113, 114). A similar relationship was found comparing urban Zulus and their rural relatives. Rural Zulus consumed 280 more calories per day (1% were from sugar) than urban Zulus (16% were from sugar). Diabetes prevalence was dramatically higher in urban than rural Zulu speakers (113, 115, 116).

### Inuit

The traditional Inuit diet was primarily animal based, including land mammals with primarily saturated fat and sea mammals with primarily polyunsaturated and monounsaturated fats. Unrefined carbohydrates made up a small fraction of their diet. In the 1960s, Inuit began to work government jobs and consumed cafeteria and sponsored food programs (117). In 1959, nomadic Inuit consumed three times as much protein as urban Inuit. Urban Inuit consumed ~50% carbohydrates, with fewer unrefined and more refined carbohydrates, and fewer fats. From 1955–1959, obesity was infrequent (<6%) (118) and diabetes was identified in no Inuit in one survey (119) and in 2/16,000 in another (120). By 2007, 15.8% of Inuit men and 25.5% of Inuit women were obese (121). In Alaska and Greenland Inuit, diabetes rates increased almost 3 times between the 1960s and 1970s. Arterial calcifications increased 5-fold in settled families who abandoned their nomadic lifestyle. The rise in obesity, diabetes, and other NCDs was associated with a transition to a more processed diet with a marked rise in total and refined carbohydrates; it is uncertain if total caloric intake changed (118, 122, 123).

### Hadza

The Hadza tribe of northern Tanzania were primarily hunter-gatherers before 1965: “nomads, getting their food by hunting various wild animals and collecting berries, tubers, baobab and wild honey” (105). The traditional diet was 60–80% plants, 20–40% meat (often lean), with honey comprising 15% of calories (124–126). After resettlement in the mid-1960s into government housing, their health declined due to infectious diseases and most resumed their hunter-gatherer lifestyle by 1979 (127). Some grew maize and millet and many consumed government-suppled foods when available (105). When diets changed, carbohydrates and refined foods increased while physical activity decreased, as most abandoned long hunting trips and their nomadic lifestyle (105). Obesity and diabetes remained rare in the Hadza before and after transition, although most retained a largely hunter-gatherer lifestyle and consumed relatively little ultra-processed food. Their lipid profiles, blood pressures and BMIs remain low compared to western populations (105, 126). Although some Hadza did not return to their hunter-gatherer lifestyle, these individuals have not been systematically studied and there is little data on their diet, lifestyle, or health.

## Discussion

Our systematic review of the dietary and lifestyle transitions of the Yemenite Jews, Tokelauns, Tanushimaru and Maasai revealed overlapping but distinct health outcomes that do not implicate total fat or SFA in NCD pathogenesis. Our data support sugar and refined carbohydrates in causing obesity, diabetes and other NCDs. Increased calories and decreased physical activity were also present in several groups. Our analysis of seven other groups (Pima and Navajo Native Americans, Aboriginal Australians, Hadza, Inuit, Natal Indians and Zulus) undergoing a diet and lifestyle transition all support that the common element associated with rising obesity and diabetes were sugar and other refined carbohydrates (Table 2). The exception was the Hadza who largely returned to their traditional diet and lifestyle and who did not develop high rates of NCDs. Data from these 11 groups were inadequate to assess the potential role of increased calories, decreased physical activity, stress, sleep disorders, medications and other factors. Although fats and SFAs were linked to obesity, diabetes, and CHD in the United States (US) and elsewhere (128), using international comparisons, rising obesity and diabetes occurred during periods when US fat and SFA consumption declined and sugar consumption increased (129, 130).

**Table 2:** Macronutrient and health outcome summary after transition (non-systematically reviewed groups).

**Groups**		**Macronutrients (% total kcal)**			**Types of fat (% total fat)**			**Types of carbohydrates (% total carbs)**			**Health measurements post transition**			
	**Calories (kcal)**	**Fat**	**Protein**	**Carb**.	**SFA**	**PUFA**	**MUFA**	**Starch**	**Fiber**	**Added sugar**	**BMI**	**Diabetes**	**Hypertension**	**Serum cholesterol (total)**
Navajo	↑	↑	-	↑	↑	↑	-	↓	↓	↑	↑	↑	↑	↑
Aboriginal Australians	?	↑	-	↓	↑	↑	?	↓	↓	↑	↑	↑	↑	↑
Natal Indian South Africans	-	-	-	-	?	?	?	↓	↓	↑	↑	↑	↑	↑
Urban Zulu Speakers	?	?	?	?	?	?	?	↓	↓	↑	↑	↑	↑	↑
Inuit	?	↓	↓	↑	?	↓	↓	↑	↑	↑	↑	↑	↑	↑
Hadza	?	-	-	-	?	?	?	?	?	?	-	-	-	-

*Key: ↑ increase, ↓ decrease, - neutral, ? uncertain*.

Several groups replaced SFAs from animal (Maasai, Inuit and Navajo) or plants (Tokelau) with refined carbohydrates, and often with MUFAs and PUFAs, but experienced increased obesity, diabetes and other NCDs. For Yemenite Jews, consumption of animal fat was stable while vegetable oil doubled and sugar increased nine-fold, and calories increased by 10% (50). The pre-transition Tanushimaru diet was mainly unrefined carbohydrates. Their post-transition diet was higher in fat but heart disease and cancer rates were stable while stroke rates declined. Among the 11 groups, sugar and refined carbohydrates increased markedly in all with large increases in obesity, diabetes, and NCDs. This concept is ancient. Excessive sugar and carbohydrate consumption were considered causes of obesity and diabetes in India (500–1000 BCE), and other 20^th^ century groups that transitioned from traditional diets to diets rich in sugar and refined carbohydrates: Asian Indians (131, 132), underprivileged Americans (133), and Icelanders (134), observed by hundreds of missionary physicians across the world (115, 135, 136).

The potential adverse health outcomes of SFAs were indirectly supported in 1961 by the Framingham Heart Study (FHS) finding that high cholesterol levels were a risk factor CHD, since SFAs raised total cholesterol in feeding studies (137). However, the prospective FHS diet study followed 1000 subjects and found no relationship between dietary fat nor SFA consumption and obesity, cholesterol levels, or CHD. This study was suppressed from peer-review publication (15). Decades later, the FHS director admitted in an editorial, that ‘the more saturated fat one ate, the *more calories one ate*, the lower the person's serum cholesterol…(and they) weighed the least (100, 109, 133, 138–145).”

Excess calories may have contributed to obesity and NCDs in many [e.g., Bengali Brahmans (131), Yemenite Jews (146), Tokelauns (138), Maasai (113), Pima (147)] but not all groups, as some were impoverished, consuming low calorie diets with obese and diabetic adults [e.g., Natal Indians (1600–2000 calories/day), (148, 149) Africa, (150) Trinidad, (151) Jamaica, (152) Sioux on reservations (137)] while their children were malnourished. This dual burden – obesity in mothers and underweight malnourished children – is present worldwide, affecting wealthy and poor nations (153). Some impoverished obese adults held physically demanding jobs and consumed low calorie diets with little access to healthcare and limited resources. Dual burden populations challenge the energy balance hypothesis of obesity, which implies that mothers overeat and underfeed their children (8). Unlike adults, underfed children cannot lower their metabolic rate until they have lost more than 30% of their weight (154). Adults reduce metabolic rate up to one-third to compensate for semi-starvation (128, 155–158). Mothers consuming low calorie diets rich in refined carbohydrates develop elevated insulin levels, insulin resistance, obesity and metabolic syndrome over decades (130, 159, 160).

Additional challenges to the energy balance model of obesity come from metabolic and hormonal studies (8, 9), chidren in foraging populations with higher physical activity but similar total energy expenditure as children from industrialized communities (139), prospective population studies (161, 162), overfeeding studies (140, 148). The energy balance hypothesis was not supported by the dietary-weight observations in Framingham and randomized dietary trials (161, 162). Even in prospective studies, caloric estimates are imprecise and caloric changes inconsistently correlate with weight changes. In overfeeding studies, healthy subjects with stable activity levels consumed a ~90% fat diet of 5,975 calories daily for five weeks with stable or lower weight (140, 148). Moderate to high calorie low-carbohydrate diets can reduce weight and diabetic symptoms (6, 148), sometimes yielding greater weight loss than lower calorie high carbohydrate diets (141). Discrepancies between calorie consumption and weight may reflect inaccurate calorie counts, differences in physical activity, refutation of the energy balance hypothesis, or some combination of these. In the Women's Health Initiative (WHI), 20,000 women consumed a “heart-healthy” diet with 114 fewer daily calories than 29,000 women on a conventional American diet. After 7.5 years, their weights were nearly identical; energy balance predicts that 328,000 excess calories should translate to more than 80 pounds of fat, but neither BMI nor waist circumference differed between the groups. The serial NHANES data on caloric consumption and weight, as well as the FHS diet study, failed to support energy balance.

Since obesity, diabetes and cardiovascular disease were rare among early-mid 20^th^ century Japanese consuming very high carbohydrate/low fat diets, Joslin, Himsworth and others considered fats pathogenic and all carbohydrates non-pathogenic. They assumed that all carbohydrates were “equivalent” (142–144, 163) since starch is digested to simple sugars. The safety of a high carbohydrate, high sucrose diet for diabetics was promoted by the American Diabetes Association up to 2008 (145). Early 20^th^ century epidemiological data strongly linked sugar consumption with obesity and diabetes within and between populations (164), and experimental and clinical data showed that low carbohydrate diets reversed diabetic symptoms in animals and humans (165). In rhesus monkeys, in six months, a high sucrose diet produced insulin resistance, central obesity, dyslipidemia, and some developed type 2 diabetes (166). Remarkably, Joslin's first diabetes textbook (1916) used a single case as an exemplar of diabetes etiology. This 49 year old overweight man had a strong family history of diabetes and ate large amounts of candy. After gallstone surgery, friends ‘sent him much candy, which he ate' and within a month, was diabetic (156). Joslin reported (1946) that tooth destruction often preceded diabetes onset, yoking sugar, tooth decay and diabetes (157). The first report of the now epidemic nonalcoholic fatty liver disease was a 41-year old obese man who drank >20 Coca-Cola bottles daily (158). Disentangling high caloric and high sugar intake in obesity and diabetes is a recurrent challenge, exemplified by the Pima (147, 167). Duration of exposure to high sugar diets may be critical. Yemenites had been in Israel far longer than the Tokelauans were in New Zealand, consistent with Campbell's incubation period of >20 years for diabetes to emerge in populations consuming >70 pounds of sugar yearly (51). By contrast, the Pima had obesity more than 50 years before diabetes rapidly increased, although data on obesity from 1900 to 1970 is sparse. Among the four systematically reviewed populations, the two with the largest increases in sugar consumption – Tokelau (650%) and Yemenites (600%) - had the highest increase in diabetes (~3- and ~40-fold, respectively). Similar patterns were seen in the Aboriginal Australians, Pima, Navajo, Natal Indians, Zulu speakers, and Inuit.

Different metabolic and health effects of sugars and refined vs. unrefined carbohydrates emerged over the 20^th^ century and are critical to understand NCD pathogenesis. The glucose in starch and the fructose/glucose in sucrose have different metabolic fates and different effects on hormones, lipogenesis and inflammation. Refined and unrefined carbohydrates have different glycemic indices, temporal effects on insulin release, nutritional elements (e.g., more fiber, protein and minerals in unrefined), and health effects (e.g., appendicitis and constipation with refined). Populations consuming >80% unrefined carbohydrates had very low rates of obesity or NCDs (135). Studies in experimental animals and human populations consuming diets rich in sugar and refined carbohydrates developed the most prevalent American hyperlipidemia (elevated VLDL and triglycerides and low HDL) (168, 169), metabolic syndrome (hyperinsulinemia, insulin resistance, abdominal obesity, hypertension, hyperglycemia, non-alcoholic fatty liver disease) (4), T2D and other NCDs (159, 166, 170, 171). Together, these findings support that sugar and refined carbohydrates contribute to or cause NCDs.

The shift toward a more industrialized diet began in Europe and North America and spread worldwide (153, 160, 172, 173). Key drivers of this shift include lower cost, higher calorie density, convenience, urbanization and other demographic shifts (e.g., working mothers and pressure for ready-to-eat meals), influences of the food industry advertise highly profitable/low quality foods (e.g., soft drinks, sugary cereals), and the addictive nature of these highly processed foods (42, 173, 174). Affluent countries and affluent individuals in poor countries often have greater access to more expensive, healthier minimally processed foods such as fruit, vegetables, nuts, and fish.

Our study has limitations. Foremost was variability in dietary surveys (pre- to post-transition and across groups), assessment across seasons, and among different population subgroups and gender, and degree of detail regarding macronutrient components (e.g., SFA, MUFA, PUFA; unrefined vs. refined carbohydrates). Studies used different criteria and methods for how they measured the diet and physical activity between these populations, making it difficult to draw concrete conclusions. Diets also varied between individuals which studies attempted to correct for in various ways, making the data less standardized. Health outcomes data was often limited for the pre-transition period. Recall bias and filling-in are additional confounds prevalent in all studies. For example, the Yemenite diet was recalled after a decade or more. Obese individuals may underestimate calorie intake during recall studies than lean individuals (175). Post-transition diets changed over time; for example, decreased fiber, increasing fructose in fruits and fat in corn fed animals. Game meat has different SFA/MUFA/PUFA profiles than store-bought meat. Information on animal parts (e.g., offal vs. muscle) consumption was rarely specified. Few studies accounted for seasonal food availability, which influenced macronutrient composition and calorie intake. The pre- to post-transition interval varied among groups. The detail, rigor and validation of the assessments varied widely, although these populations were among the most rigorously studied. Further, carefully designed prospective dietary surveys have limited validity. Other factors, including physical activity, cigarette smoking, stress, sleep, comorbid psychiatric disease, and medications with positive (e.g., antihypertensive) or negative (e.g., antipsychotics causing metabolic syndrome) were infrequently and informally assessed. Rarely did a study include all of these factors together in a standardized way. Thus, the reduced stroke rate in Tanushimaru associated with higher fat intake may be due to the increased use of anti-hypertensive medications. Importantly, some groups underwent a physical transition (Yemenite, Tokelau, Maasai) while others did not (Tanushimaru). Different environmental exposures were not accounted for in these studies. We only systematically studied four among many populations that underwent a nutrition and lifestyle transition. However, among the six other groups that had a dietary and lifestyle transition (Pima, Inuit, Rural Chinese, Natal Indians, Aboriginal Australians), all had negative health outcomes and increased sugar consumption. Finally, many studies only assessed men or specific age ranges, excluding most of the population. Therefore, data from these transitional populations cannot definitively determine the role of dietary factors in health outcomes.

## Conclusion

Four geographically and culturally diverse populations with different diets and lifestyles underwent a nutrition and lifestyle transition. The development of obesity, T2D and other NCDs was associated with a marked increase in sugar and refined carbohydrates and a more modest increase total caloric intake. The dietary transition of three populations we studied included a significant reduction in SFAs and in two, total fat consumption. All three developed NCDs despite the reductions in SFAs and fats. The Tanushimaru, our only population without increased sugar and refined carbohydrate consumption, had stable caloric intake with increased fat consumption: their health improved, with reduced breast cancer and stroke (possibly due to antihypertensive medications). These findings were supported by data from seven additional populations. In the ten populations with high rates of post-transition NCDs, increased sugar and refined carbohydrates were the common factor. Increased calories and decreased activity was present in many but not all, and may contribute to NCDs. Neither SFA nor total fat consumption were associated with NCDs. Although many other potential contributory factors to healthy outcomes were not adequately assessed (e.g., stress, medications, sleep), similar observations among scores of other populations who underwent a nutrition and lifestyle transition implicate sugar and refined carbohydrates as key dietary factors.

We confirm that we have read the Journal's position on issues involved in ethical publication and affirm that this report is consistent with those guidelines.

## Data Availability Statement

The original contributions presented in the study are included in the article/Supplementary Material, further inquiries can be directed to the corresponding author/s.

## Author Contributions

MP, JD, MD, JL, CG, SW, JL, and DF contributed to data analysis, writing and reviewing the manuscript. TR contributed to data and reviewing the manuscript. OD contributed to study design, data analysis, writing and reviewing the manuscript.

## Funding

This work was supported by the Finding A Cure for Epilepsy and Seizures (FACES) organization affiliated with NYU Langone Health and the Comprehensive Epilepsy Center. The foundation had no role in study design, data collection and analysis, decision to publish, or preparation of the manuscript.

## Conflict of Interest

OD Research funding, Novartis, PTC Therapeutics, Marinus Pharmaceuticals, GW Pharmaceuticals; Zogenix; Equity interest, Rettco, Pairnomix, Tilray, Egg Rock Holdings; California Cannabis Enterprises. DF receives salary support for consulting and clinical trial related activities performed on behalf of The Epilepsy Study Consortium, a non-profit organization, receives no personal income for these activities. NYU receives a fixed amount from the Epilepsy Study Consortium toward DF's salary. Within the past two years, The Epilepsy Study Consortium received payments for research services performed by DF from: Axcella, Biogen, Cerevel, Crossject, Engage Pharmaceuticals, Eisai, Pfizer, SK Life Science, Xenon, and Zynerba. He has also served as a paid consultant for Eisai and Neurelis Pharmaceuticals. He has received travel support from Medtronics, Eisai and the Epilepsy Foundation. He receives research support from the CDC, NINDS, Epilepsy Foundation, Empatica, Epitel, UCB, Inc. and Neuropace unrelated to this study. He serves on the scientific advisory board for Receptor Life Sciences. He holds equity interests in Neuroview Technology and Receptor Life Sciences. The remaining authors declare that the research was conducted in the absence of any commercial or financial relationships that could be construed as a potential conflict of interest.

## Publisher's Note

All claims expressed in this article are solely those of the authors and do not necessarily represent those of their affiliated organizations, or those of the publisher, the editors and the reviewers. Any product that may be evaluated in this article, or claim that may be made by its manufacturer, is not guaranteed or endorsed by the publisher.

## Abbreviations

BMI: Body mass index
CHD: Coronary heart disease
CVD: Cardiovascular disease
LDL: Low density lipoprotein
MUFA: Monounsaturated fatty acids
NCD: Non-communicable chronic diseases
PUFA: Polyunsaturated fatty acids
RCT: Randomized control trial
SFA: Saturated fatty acids
T2DM: Type II diabetes mellitus.
