# Supplementary material for: Dietary Transitions and Health Outcomes in Four Populations – Systematic Review
Source: Front Nutr. 2022 Feb 9;9:748305. doi: 10.3389/fnut.2022.748305 (PMC8892920; doi:10.3389/fnut.2022.748305)
Supplement: Supplementary file 1 [file Table_1.docx]

*Supplemental Table 1* Availability of data on various factors.

| Population | Transitional Period | Macronutrients | BMI | Diabetes | Hypertension | Physical Activity | Smoking | Alcohol |
| --- | --- | --- | --- | --- | --- | --- | --- | --- |
| Yemenite | Pre-Transition | ✓ | ✓ | ✓ | X | ✓ | ✓ | ✓ |
|  | Post-Transition | ✓ | ✓ | ✓ | ✓ | ✓ | ✓ | ✓ |
| Tokelau | Pre-Transition | ✓ | ✓ | ✓ | ✓ | ✓ | ✓ | X |
|  | Post-Transition | ✓ | ✓ | ✓ | X | X | X | X |
| Tanushimaru | Pre-Transition | ✓ | ✓ | X | X | ✓ | ✓ | X |
|  | Post-Transition | ✓ | ✓ | X | X | ✓ | ✓ | ✓ |
| Maasai | Pre-Transition | ✓ | ✓ | ✓ | X | ✓ | X | ✓ |
|  | Post-Transition | ✓ | ✓ | X | X | ✓ | ✓ | ✓ |
