# Supplementary material for: Dietary Transitions and Health Outcomes in Four Populations – Systematic Review
Source: Front Nutr. 2022 Feb 9;9:748305. doi: 10.3389/fnut.2022.748305 (PMC8892920; doi:10.3389/fnut.2022.748305)
Supplement: Supplementary file 2 [file Data_Sheet_1.PDF]

MAASAI	Population Cohort	Methods	Pre-transition				Post-transition			
			Dietary Findings	BMI	Lifestyle	Major outcomes	Dietary Findings	BMI	Lifestyle	Major outcomes
Christensen DL, Eis J, Hansen AW, et al. Obesity and regional fat distribution in Kenyan populations: impact of ethnicity and urbanization. *Ann Hum Biol*. 2008 Mar-Apr 2008;35(2):232-49. doi:10.1080/03014460801949870	Pre-transition: 172 Males and 193 Females Maasai in rural Kenya, lived in villages (manyattas) in 2005-2006<br>Post-transition: 7 Males Maasai in urban Kenya (Nairobi) in 2005-2006	Observational study. Data were collected from anthropometric measures, BMI, arm fat area (AFA), and arm muscle area (AMA).	-	Males: 21.0 +/- 4.7<br>Females: 22.3 +/- 4.7	-	-	-	Males: 23.3 +/- 4.4	-	-
Christensen DL, Faurholt-Jepsen D, Boit MK, et al. Cardiorespiratory fitness and physical activity in Luo, Kamba, and Maasai of rural Kenya. *Am J Hum Biol*. 2012 Nov-Dec 2012;24(6):723-9. doi:10.1002/ajhb.22303	Pre-transition: 163 Males and 178 Females Maasai in rural Kenya in 2005<br>Post-transition: N/A	Observational study. Data were collected on physical activity energy expenditure (PAEE) and cardio-respiratory fitness (CRF) by administering a step test, monitoring heart rate data, and a performing a blood test.	-	Males: 21.1 (95% CI: 20.5-21.6)<br>Females: 22.7 (95% CI: 22.1-23.4)	Males: physical activity energy expenditure (PAEE): 78.0 kJ/day*kg (95% CI: 74.2-81.7); cardio-respiratory fitness (CRF): 43.1 mL O2/min*kg (95% CI: 42.1-44.1)<br>Females: PAEE: 74.5 kJ/day*kg (95% CI: 70.1-78.9); CRF: 38.9 mL O2/min*kg (95% CI:37.8-39.9)<br>Physical activity levels among rural Maasai (and Kamba) were the highest in the group.	-	-	-	-	-
Christensen DL, Friis H, Mwaniki DL, et al. Prevalence of glucose intolerance and associated risk factors in rural and urban populations of different ethnic groups in Kenya. *Diabetes Res Clin Pract*. Jun 2009;84(3):303-10. doi:10.1016/j.diabres.2009.03.007	Pre-transition: 172 Males and 193 Females rural Maasai in 2005-2006<br>Post-transition: 7 Males in urban Maasai in 2005-2006	Observational study. Data were collected from anthropometric measures, ultrasonography (fat thicknesses), physical activity and fitness measurements, and questionnaire data.	Subsists on mainly animal husbandry and maize; had a relatively lower dietary glycemic load compared to the other groups	Had the highest fat accumulation expressed as BMI, visceral and abdominal subcutaneous fat thickness, WC and AFA with increasing age compared to the Luo and Kamba (no quantitative data)	-	Males: 5.2% with Glucose intolerance (diabetes + IGT)<br>Females: 7.8% with Glucose Intolerance (diabetes + IGT)	-	-	-	-
Day J, Bailey A, Robinson D. Biological variations associated with change in lifestyle among the pastoral and nomadic tribes of East Africa. *Ann Hum Biol*. 1979 Jan-Feb 1979;6(1):29-39. doi:10.1080/03014467900003341	Pre-transition: 39 tribal Males living in Tembo Gate, studied in August 1977<br>Post-transition: 22 non-tribal Males living Keekorok Game Lodge, studied in August 1977	Observational study. Data were collected from anthropometric measures, blood pressure measurements, plasma cortisol estimations, cholesterol, and triglyceride levels.	Milk, supplemented with ox blood and meat, and hence is rich in cholesterol.	Males: Height: 174.78 +/- 6.9 cm; weight: 58 +/- 6.13 kg	More active than "non-tribal" members	Males: SBP: 108.95 +/- 10.91 mmHg; DBP: 70.26 +/- 8.22 mmHg; Cholesterol: 193. 42 +/- 35.85 mg/dL; Triglyceride: 125.68 +/- 54.08 mg/dL; Fibrinogen: 12.25 +/- 5.27 mg/dL	Cholesterol replaced with carbohydrates.	Males: Height: 170.51 +/- 5.59 cm; weight: 58.55 +/- 4.88 kg	Less active than "tribal" members, but considerably very active by Western standards	Males: SBP: 117.50 +/- 5.96 mmHg; DBP: 72.75 +/- 7.69 mmHg; Cholesterol: 187.25 +/- 35.85 mg/dL; Triglyceride: 123.15 +/- 52.94 mg/dL; fibrinogen: 15.53 +/- 7.55 mg/dL
Day J, Carruthers M, Bailey A, Robinson D. Anthropometric, physiological and biochemical differences between urban and rural Maasai. Atherosclerosis. 1976/03/01/ 1976;23(2):357-361. doi:https://doi.org/10.1016/0021-9150(76)90109-X	Pre-transition: 27 Males from a rural group in 1975<br>Post-transition: 26 Males who had been living in cities for at least 10 years in 1975	Observational study. Data were collected from anthropometric measures, blood pressure measurements, cholesterol, triglyceride, and fibrinogen.	Staple food of milk, supplemented with blood and meat, with a cholesterol content similar to American and Western European diets.	Males: Height: 172.27 +/- 7.06 cm; weight: 55.47 +/- 9.76 kg	More active than the urban subjects.	Males: SBP: 114.07 +/- 24.89 mmHg; DBP: 65.33 +/- 14.62 mmHg; Cholesterol: 160.19 +/- 46.93 mg/dL; Triglyceride: 144.04 +/- 74.14 mg/dL; Fibrinogen: 206.43 +/- 49.99 mg/dL	Consumed more carbohydrates than rural group.	Males: Height: 173.21 +/- 7.45 cm; weight: 66.39 +/- 8.81 kg	Tended to be engaged in sedentary occupations.	Males: SBP: 110.96 +/- 14.56 mmHg; DBP: 70.00 +/- 12.65 mmHg; Cholesterol: 202.88 +/- 47.33 mg/dL; Triglyceride: 158.85 +/- 68.12 mg/dL; Fibrinogen: 184.35 +/- 35.00 mg/dL
Galvin KA, Beeton TA, Boone RB, BurnSilver SB. Nutritional Status of Maasai Pastoralists under Change. *Hum Ecol Interdiscip J*. 2015 2015;43(3):411-424. doi:10.1007/s10745-015-9749-x	Pre-transition: 219 Males and 315 Females from 0-18+y/o from pastoral Maasai, and 96 Males and 130 Females of whom are adults in 2000<br>Post-transition: N/A	Observational study. Data were collected on anthropometric measures, nutritional data, and diet recall data.	A combination of pastoral foods (milk and meat) and non-pastoral foods (tea leaves, sugar, salt, maize meal, fat, rice, beans, potatoes, tomatoes, onions, Sukuma, wheat flour, chicken and combinations thereof)	-	-	Males: adults: 4.1% obese, wasted/underweight: 51%, malnourished: 52%, stunted: 0%<br>Females: adults: 4.6% obese, wasted/underweight: 559%, malnourished: 1.5%, stunted: 2.3%	-	-	-	-
Hansen AW, Christensen DL, Larsson MW, et al. Dietary patterns, food and macronutrient intakes among adults in three ethnic groups in rural Kenya. *Public Health Nutr*. Sep 2011;14(9):1671-9. doi:10.1017/S1368980010003782	Pre-transition: 172 Males and 190 Females Maasai from rural Kenya in 2005<br>Post-transition: N/A	Observational study. Data were collected from 24h food intake recalls, anthropometric measures, and socioeconomic surveys.	Males: Total energy: 8400 kJ/d, protein: 71.3 g/d (14.1%E), fat: 68.2 g/d (30.2%E), carbohydrate: 273 g/d (55.7%E)<br>Females: Total energy: 6700 kJ/d, protein: 50.8 g/d (12.8%E), fat: 47.0 g/d (26.2%E), carbohydrate: 240 g/d (61.0%E)<br><br>Milk and milk products were important food items	Males: 21.0 (95% CI: 20.3-21.7)<br>Females: 22.3 (95% CI: 21.6-23)	-	Males: BMI >/= 30 (obese): 4.1%, BMI 25-29.9 (overweight): 9.9%, BMI 18.5-24.9: 55.8%, BMI < 18.5 (underweight): 30.2%<br>Females: BMI >/= 30 (obese): 10.0%, BMI 25-29.0 (overweight): 7.9%, BMI 18.5-24.9: 63.2%, BMI < 18.5 (underweight): 19%	-	-	-	-
Homewood K. Pastoralist production systems and climate change. *Climate change and World Food security*. Springer; 1996:505-524.	Pre-transition: 1960's Kenyan and Tanzanian Maasai, "pre-development"<br>Post-transition: 1980's Kenyan and Tanzanian Maasai, "post-development"	Comparative study of past published studies.	-	Tanzanian 1989:<br>Males: 21.0<br>Females: Adult: 22.4, 19-30y/o: 22.9	-	-	Tanzanian 1981-1983: Milk/milk products: 34% +/- 25.3%, meat: 10% +/- 7.5%, maize: 53% +/- 21.8%, other: 4%<br>Kenyan 1981-1983: Milk/milk products: 48% +/- 32.8%, meat: 8% +/- 12.0%, maize: 26% +/- 26.3%, other: 19%	Tanzanian 1989:<br>Males: 19.7 +/- 2.3<br>Females: Adult: 19.1 +/- 2.3; 18-29y/o: 19.7 +/- 2.0	-	-
Knoll N, Kuhnt K, Kyallo FM, Kiage-Mokua BN, Jahreis G. High content of long-chain n-3 polyunsaturated fatty acids in red blood cells of Kenyan Maasai despite low dietary intake. *Lipids Health Dis*. Aug 2011;10:141. doi:10.1186/1476-511X-10-141	Pre-transition: N/A<br>Post-transition: 6 Males and 20 Females Kenyan Maasai in 2007	Observational study. Data were collected on food intake surveys and blood draws.	-	-	-	-	Males: energy intake: 9.5 MJ; carbohydrates: 303 g; protein: 73g; fat: 74g<br>Females: energy intake: 7.0 MJ; carbohydrates: 223g; protein: 54g; fat: 55g<br>Overall: energy intake: 7.6 MJ +/- 2.1 MJ; carbohydrates: 56.2%E +/- 9.4%; protein: 13.3%E +/- 2.0%E; fat: 30.5%E +/- 7.9%E<br>Dairy products: 41.1%E +/- 20.7%E; Meat and suet: 0.6%E +/- 1.9%E; plant-derived foods: 58.3%E +/- 20.7%E		Males: Alcohol: 0g<br>Females: Alcohol: 0g<br>More sedentary, market-orientated lifestyle	Males (n=4 agreed to blood draw): Total cholesterol: 4.3 mmol/L +/- 0.5 mmol/L; LDL: 2.8 mmol/L +/- 0.6 mmol/L; HDL: 0.9 mmol/L +/- 0.1 mmol/L<br>Females (n=14 agreed to blood draw): Total cholesterol: 4.3 mmol/L +/- 1.1 mmol/L; LDL: 2.8 mmol/L +/- 0.9 mmol/L; HDL: 1.1 mmol/L +/- 0.2 mmol/L
Lee JC, Westgate K, Boit MK, et al. Physical activity energy expenditure and cardiometabolic health in three rural Kenyan populations. *Am J Hum Biol*. 01 2019;31(1):e23199. doi:10.1002/ajhb.23199	Pre-transition: N/A<br>Post-transition: 161 Males and 170 Females rural Maasai in 2005	Observational study. Data were collected from anthropometric measures, blood pressures, glucose challenge test, health assessment questionnaires, 24-hour dietary intake recalls, and physical activity measurements.					Males: total fat intake: 2670 +/- 1080 kJ/day<br>Females: total fat intake: 1820 +/- 763 kJ/day	Males: Average BMI: 21.0 +/- 4.8<br>Females: 22.5 +/- 4.8	Males: smoking status: 15.8% yes (95% CI: 10.9-22.4); alcohol consumption: 14.9% yes (95% CI: 10.2-21.3); physical activity energy expenditure (PAEE): 76.6 +/- 25.8 kJ/kg/day<br>Females: smoking status: 10.8% yes (95% CI: 6.9-16.6); alcohol consumption: 3.5% yes (95% CI: 1.6-7.7); physical activity energy expenditure (PAEE): 73.5+/- 23.1 kJ/kg/day	Males: Average SBP: 124 +/- 14.6 mmHg; average DBP: 77 +/- 9.8 mmHg; total cholesterol: 4.0 +/- 1.1 mmol/L; HDL: 1.1 +/- 0.32 mmol/L; LDL: 2.4 +/- 0.9 mmol/L; blood glucose (fasting): 4.4 +/- 1.0 mmol/L; blood glucose (30 min): 6.3 +/- 1.5 mmol/L; blood glucose (120 min): 4.5 +/- 1.7 mmol/L; insulin (fasting): 24 pmol/L (IQR: 19 pmol/L)<br>Females: Average SBP: 116 +/- 13.2 mmHg; DBP: 73 +/- 9.3 mmHg; total cholesterol: 3.8 +/- 0.9 mmol/L; HDL: 1.2 +/- 0.3 mmol/L; LDL: 2.2 +/- 0.7 mmol/L; blood glucose (fasting): 4.6 +/- 1.3 mmol/L; blood glucose (30 min): 6.1 +/- 1.6 mmol/L; blood glucose (120 min): 5.3 +/- 1.5 mmol/L; insulin (fasting): 26 pmol/L (IQR: 21 pmol/L)
Mann GV, Shaffer RD, Anderson RS, Sandstead HH. Cardiovascular Disease in the Masai. *J Atheroscler Res*. 1964 Jul-Aug 1964;4:289-312. doi:10.1016/s0368-1319(64)80041-7	Pre-transition: 400 Males and 'some' additional Females/children in South Maasailand (rural) in 1962<br>Post-transition: N/A	Observational study. Data were collected from anthropometric measures, blood pressures, medical history, ECG, serum cholesterol levels, and dietary intakes.	Consumed high levels of milk (protein and fat)	Males: Average height for 14-19y/o: 165.6 +/- 1.42 cm, 20-24y/o: 171.6 +/- 0.74 cm, 25-29y/o: 173.3+/-1.27 cm, 30-43y/o: 170.9 +/- 0.71 cm, 44-55y/o: 172.0 +/- 0.56 cm, 55+ y/o: 160.5 +/- 3.89 cm; Average weight for 14-19y/o: 46.2 +/- 1.2 kg, 20-24y/o: 56.1 +/- 0.72 cm, 25-29y/o: 60.8+/- 1.7 cm, 30-43y/o: 57.3 +/- 0.69 cm, 44-55y/o: 58.8 +/- 0.63 cm, 55+ y/o: 48.4 +/- 1.5 cm	-	Males: Cholesterol of 14-19y/o: 114.7 +/- 5.62 mg/dL, 20-24y/o: 114.8 +/- 2.60 mg/dL, 25-29y/o: 130.2 +/- 5.23 mg/dL, 30-43y/o: 129.9 +/- 3.57 mg/dL; 44-55y/o: 129.6 +/- 2.53 mg/dL, 55+: 113.7 +/- 16.36 mg/dL; all ages: 124.6 +/- 1.55 mg/dL; Total % of heart disease: 3.36%; SBP greater than or equal to 160 mmHg: 1.5%; DBP than or equal to 100 mmHg: 1.3%	-	-	-	-
Nestel P. A society in transition: Developmental and seasonal influences on the nutrition of Maasai women and children. UN; 1986.	Pret-transition: Group ranch Mbirikani 190 children and 83 adults in 1982-1983<br>Post-transition: Group ranches Olkarkar and Merueshi 198 children and 108 adults in 1982-1983	Observational study. Data were collected over two years from anthropometric measures, 24-hour dietary recall, and socioeconomic information.	Protein: 14%E, fat: 35%E, carbohydrate: 51%E<br><br>Food derived from own livestock (milk, meat, blood): Mbirikani: milk and milk products: 31%E, meat and animal fats: 12%E, purchased foods (maize, sugar, other): 56%E	-	Alcohol: 0%E	-	Females: Protein: 14%E, Fat: 46%E, Carbohydrate: 39%E<br><br>Food derived from own livestock (milk, meat, blood): milk and milk products (milk and butter): 64%E, meat and animal fats: 4%E, purchased foods (maize, sugar, other): 32%E	-	Alcohol: 1%E	-
Nestel PS. Food intake and growth in the Maasai. *Ecology of Food and Nutrition*. 1989;23(1):17-30.	Pre-transition: 61 Females (non-pregnant and non-lactating) in rural Maasai in 1982-1983<br>Post-transition: N/A	Observational study. Data were collected from anthropometric measures, 24-hour dietary recall, and samples of principle foods.	Females: Dairy products (milk and butter): 42.4 +/- 29.4%E, meat and animal fats: 9.2 +/- 12.4%E, purchased foods (maize, sugar, other): 48.4 +/- 12.1%E, Protein: 14.8 +/- 7.3%E; fat: 35.8 +/-14.1%E, carbohydrate: 48.4 +/- 18.4%E, alcohol: 0.9 +/- 4.8	-	Females: Alcohol: 0.9 +/- 4.8%E	-	-	-	-	-
Nestel P, Geissler C. Lipid intakes of Maasai women and children. *Ecology of food and nutrition*. 1993;29(2):155-165.	Pre-transition: 121 Females in rural Maasai in 1982-1983<br>Post-transition: N/A	Observational study: Data were collected for a 24-hour dietary recall.	Females: Total energy intake (kcal): 1329 +/- 19; fat (g): 53.6 +/- 1.1; %E from fat: 37.8 +/- 0.7; Saturated fat (proportion of total fatty acids, %): 68.1 +/- 0.53; monounsaturated fat: 28.0 +/- 0.29; polyunsaturated fat: 3.5 +/- 0.22; total cholesterol (mg): 219.1 +/- 5.2	-	-	-	-	-	-	-
Njelekela M, Ikeda K, Mtabaji J, Yamori Y. Dietary habits, plasma polyunsaturated fatty acids and selected coronary disease risk factors in Tanzania. *East Afr Med J*. Nov 2005;82(11):572-8. doi:10.4314/eamj.v82i11.9412	Pre-transition: 21 Males and 11 Females pastoralist Maasai in Monduli in 1998<br>Post-transition: N/A	Observational study. Data were collected from anthropometric measures, food intake, plasma fatty acid analysis, and HBA1c levels.	Fish: <1 day/week-96.9%, 1-2 days/wk-3.1%, >2 days/wk-0%;<br>Meat: <1 day/week-50%, 1-2 days/wk-9.4%, >2 days/wk-40.6%;<br>Green vegetables: <1 day/week-50%, 1-2 days/wk-21.9%, >2 days/wk-40.6%;<br>Coconut milk: <1 day/week-96.9%, 1-2 days/wk-0%, >2 days/wk-3.1%;<br>Fruits: <1 day/week-78.1%, 1-2 days/wk-18.8%, >2 days/wk-3.1%;<br>Whole milk: <1 day/week-43.8%, 1-2 days/wk-3.1%, >2 days/wk-53.1%	Males: 21.2 +/- 0.8<br>Females: 21.5 +/- 1.9	-	Males: SBP (mmHg): 117.7 +/- 3.2; DBP (mmHg): 66.9 +/- 2.3; Total Cholesterol (mmol/L): 4.7 +/- 0.4; LDL (mmol/L): 3.1 +/- 0.3; HDL(mmol/L): 1.1 +/- 0.1; HBA1c (%): 5.7 +/- 0.1<br>Females: SBP (mmHg): 119.4 +/- 5.3; DBP (mmHg): 71.9 +/- 4.2; Total Cholesterol (mmol/L): 5.8 +/- 0.4; LDL (mmol/L): 4.0 +/- 0.3; HDL(mmol/L): 1.1 +/- 0.1; HBA1c (%): 5.7 +/- 0.1	-	-	-	-
Njelekela M, Kuga S, Nara Y, et al. Prevalence of obesity and dyslipidemia in middle-aged men and women in Tanzania, Africa: relationship with resting energy expenditure and dietary factors. J Nutr Sci Vitaminol (Toky/o). Oct 2002;48(5):352-8. doi:10.3177/jnsv.48.352	Pre-transition: 59 Males and 87 Females pastoralist Maasai in Monduli in 1998<br>Post-transition: N/A	Observational study. Data were collected from anthropometric measures, food intake, plasma fatty acid analysis, and HBA1c levels.	Males: Green vegetables (d/wk): 1.1 +/- 1.7; Coconut milk (d/wk): none; whole milk (mL/d): 1218.6 +/- 1670.0; Fish (d/wk): none; meat (d/wk): 2.0 +/- 2.4<br>Females: Green vegetables (d/wk): 1.7 +/- 2.4; Coconut milk (d/wk): 0.1 +/- 0.8; whole milk (mL/d): 785.1 +/- 841.4; Fish (d/wk): 0.1 +/- 0.4; meat (d/wk): 2.5 +/- 2.2	Males: 20.7 +/- 3.2; Obesity (BMI >/= 30): 1.7%<br>Females: 21.0 +/- 5.0; Obesity: F: 10.7%	Semi-nomadic pastoralists that tend large herds of cattle and not involved in any agricultural activities; they are lean and tall and have an active lifestyle;<br>REE (Resting energy expenditure, kcal/min/kg): Males: 0.024 +/- 0.006; Fenakes: 0.020 +/- 0.004	Males: Total Cholesterol (mmol/L): 5.1 +/- 1.5; Triglycerides (mmol/L): 1.9 +/- 1.5; LDL (mmol/L): 3.7 +/- 1.3; HDL(mmol/L): 1.1 +/- 0.4; Dyslipidemia: 22.6%<br>Females: Total Cholesterol (mmol/L): 5.3 +/- 1.2; Triglycerides (mmol/L): 1.7 +/- 0.8; LDL (mmol/L): 3.7 +/- 1.1; HDL(mmol/L): 1.3 +/- 0.4; Dyslipidemia: 9.0%	-	-	-	-
Oiye S, Simel JO, Oniang'o R, Johns T. The Maasai food system and food and nutrition security. *Indigenous Peoples' Food Systems: The Many Dimensions of Culture, Diversity and Environment for Nutrition and Health FAO*. 2009:231-249.	Pre-transition: N/A<br>Post-transition: Females from 120 Maasai households in rural Kenya in 2004-2005 during drought season	Observational study. Data were collected from key informant interviews, focus group discussions, micronutrient analysis of herbs and fruits, and food sample collection and identification.	-	-	-	-	Total Energy (Kcal): 1623, Protein (g): 126 g<br>Mainly depend on foods produced by others now, with diminished intake of milk, meat, blood, and herbs, and seasonal fruits during droughts	-	-	-
Robinson D, Williams P, Day J. High-density-lipoprotein cholesterol in the Maasai of East Africa: a cautionary note. *Br Med J*. May 1979;1(6173):1249. doi:10.1136/bmj.1.6173.1249	Pre-transition: 37 tribal Maasai Males in southern Kenya in 1977<br>Post-transition: 20 non-tribal Maasai Males in southern Kenya in 1977	Observational study. Data were collected from cholesterol levels.	-	-	-	Males (combined tribal with non-tribal): Total cholesterol levels (mmol/L:): 4.77 +/- 0.81, HDL (mmol/L): 1.05 +- 0.31; HDL to TC ratio (%): 22.63 +/- 7.71	-	-	-	Males (combined tribal with non-tribal): Total cholesterol levels (mmol/L:): 4.77 +/- 0.81, HDL (mmol/L): 1.05 +- 0.31; HDL to TC ratio (%): 22.63 +/- 7.71
Talle A. Ways of milk and meat among the Maasai: gender identity and food resources in a pastoral economy. *Ways of milk and meat among the Maasai: gender identity and food resources in a pastoral economy*. 1990:73-92.	Pre-transition: Pastoral Maasai of Kenya who subsists on producing their own foods and goods.<br>Post-transition: Pastoral Maasai of Kenya that have become sedentary, with market exchange, cash transactions, and the consumption of non-pastoral foods.	Literature review. Data were collected from past studies.	Mainly milk, meat, and blood; honey beer also drunk at formal gatherings; vegetarian foods (berries, fruits, plants, and roots) collected and eaten by women and children; crops (especially maize) consumed by every/one except for the warriors	-	-	-	Becoming increasingly dependent on purchased foodstuffs, majority of which is vegetable products; diet increasingly includes tea, sugar, cooking fat, potatoes, rice, and vegetables	-	-	-
