# Supplementary material for: Dietary Transitions and Health Outcomes in Four Populations – Systematic Review
Source: Front Nutr. 2022 Feb 9;9:748305. doi: 10.3389/fnut.2022.748305 (PMC8892920; doi:10.3389/fnut.2022.748305)
Supplement: Supplementary file 3 [file Data_Sheet_2.PDF]

| TOKELAU | Population Cohort | Methods | Pre-transition | | | | Post-transition | | | |
|---|---|---|---|---|---|---|---|---|---|---|
| | | | Dietary Findings | BMI | Lifestyle | Major outcomes | Dietary Findings | BMI | Lifestyle | Major outcomes |
| Davidson F. The Tokelau Island migrant study: atoll diet. *New Zealand Medical Journal*. 1976;83(557):89-89 | Pre-transition: Tokelauan families of all ages on the island of Fakafako in 1968.<br>Post-transition: N/A | Longitudinal study. Data were collected through a survey of food intake given to each household. | 1930 kcal, Carbs 35% E, Protein 12% E, Fats 52%<br>Consumed high levels of coconut, drinking nut, sap, oil, fish, breadfruit, and taro and low levels of milk, milk products, vegetables, and eggs. | - | Males: food gatherers, active until late middle age.<br>Females: perform mostly household chores, sedentary lifestyle. | - | - | - | - | - |
| Harding W. The diet of Tokelau Island migrants in New Zealand. *New Zealand Medical Journal*. 1976;83(557):89-89. | Pre-transition: N/A<br>Post-transition: Tokelau island families of all ages living in Taupo, New Zealand in 1974. All families migrated less than 10 years prior. | Cross sectional study. Data were collected by a survey of food intake in each household over a period of 7 days. All foods present in the beginning and end of the study period were measured. Daily visits were made to each home to record food purchased and eaten. | - | - | - | - | 2280 Kcal, Carbs 44% E, Protein 15% E, Fats 41% E<br>Consume high levels of fish, meat, rice, and potatoes and low levels of butter and milk. | - | - | - |
| Harding WR, Russell CE, Davidson F, Prior IAM. Dietary surveys from the Tokelau Island migrant study. *Ecology of Food and Nutrition*. 1986/11/01 1986;19(2):83-97. doi:10.1080/03670244.1986.9990951 | Pre-transition: 224 adults and children living on Fakaofo in 1968.<br>Post-transition: 246 adults and children living on Fakaofo in 1971 and 552 adults and children living on Fakaofo in 1976. | Cross sectional study. Data were collected by a survey of food intake in each household over a period of 7 days. All foods present in the beginning and end of the study period were measured. Daily visits were made to each home to record food purchased and eaten. In 1971 and 1976, methodology was modified to include quantitative 24-hour intakes for each individual member of the household. | 1930 kcal, Carbs 35% E, Protein 12% E, Fats 53%E | - | - | - | 2280 Kcal, Carbs 44% E, Protein 15% E, Fats 41% E<br>Consume high levels of fish, meat, rice, and potatoes and low levels of butter and milk. | - | - | - |
| Ostbye T, Welby TJ, Prior IA, Salmond CE, Stokes YM. Type 2 (non-insulin-dependent) diabetes mellitus, migration and westernization: the Tokelau Island Migrant Study. *Diabetologia*. Aug 1989;32(8):585-90. doi:10.1007/BF00285332 | Pre-transition: 35-74 y/o living on the island of Fakaofo in 1968 (phase 1). 448 adults were initially examined.<br>Post-transition: Re-examination was done in 1976 (phase 2) and 1982 (phase 3). | Cross-sectional study. Data were collected through surveys and diabetes was determined based on plasma glucose levels after an overnight fast. Blood pressure was recorded with a sphygmomanometer. | - | - | - | Males: 3% diabetic<br>Females: 8.7% diabetic | - | - | - | Male: 5.1% diabetic (1976), 7% diabetic (1982). Average SBP 130 mmHg. Average DBP<br>Female: 11.5% diabetic (1976), 14.3% diabetic (1982). Average SBP 132 mmHg. Average DBP 81 mmHg. |
| Prior IA, Davidson F, Salmond CE, Czochanska Z. Cholesterol, coconuts, and diet on Polynesian atolls: a natural experiment: the Pukapuka and Tokelau island studies. *Am J Clin Nutr*. Aug 1981;34(8):1552-61. doi:10.1093/ajcn/34.8.1552 | Pre-transition: Total population living in Tokelau was examined in 1968. 1972 people were examined.<br>Post-transition: Total population living in Tokelau was examined in 1971. 1792 people were examined. | Cross-sectional study. Data were collected through surveys on household consumption in 1968. Nutrient intake was analyzed in 1971 | Males: 2520 kcal, Carbs 36.3%E, Protein 12.5% E, Fats 55.7%E. Of the 55.7% energy from fat, 88% was saturated fat.<br>Females: 2100 kcal, Carbs 36% E, Protein 12% E, Fats 56%E. Of the 55.7% energy from fat, 92% was saturated fat. | - | - | - | - | - | - | - |
| Prior I. Food, nutrition and health of Maori and Pacific populations. presented at: Proceedings of the Nutrition Society of New Zealand; 1989. | Pre-transition: Tokelauan island population 1968<br>Post-transition: Tokelauan island population in 1982, Tokelauan migrants in NZ in 1974/5 | Systematic review. Data were collected by reviewing longitudinal studies following Maori and Tokelauan health following migration in 1970 carried out by the Epidemiology Unit at Wellington. | 1931 kcal, Carbs 35% E, Protein 12% E, Fats 53% E, Saturated fats 45% E | - | - | Males: 8% definite hypertension.<br>Females: 15.1% definite hypertension. | Tokelau: 1812 kcal, Carbs 43% E, Protein 13-% E, Fats 44% E, Saturated fats 36% E<br>New Zealand: 2282 kcal, Carbs 44% E, Protein 15% E, Fats 51% E, Saturated fats 21% E | - | - | - |
| Prior I, Hooper A, Huntsman J, Stanhope J, Salmond C. The Tokelau Island Migrant Study. *Population Structure and Human Variation*. Cambridge University Press; 1977:165-186. | Pre-transition: All ages living in Tokelau in 1968. 1792 people were evaluated.<br>Post-transition: All ages living in Tokelau and New Zealand in 1973 and 1974. 1567 people were evaluated in Tokelau and 1920 were evaluated in New Zealand. | Multiple cross-sectional studies conducted in phases. Data were collected through a series of surveys and a basic physical examination. | - | - | - | Males: Average SBP of 15-19 y/o 115 mmHg, 20-24 y/o 122 mmHg, 25-34 y/o 123 mmHg, 35-44 y/o 126 mmHg, 45-54 y/o 131 mmHg, 55-64 y/o 134 mmHg. Average DBP of 15-19 y/o 66 mmHg, 20-24 y/o 68 mmHg, 25-34 y/o 71 mmHg, 35-44 y/o 77 mmHg, 45-54 y/o 78 mmHg, 55-64 y/o 78 mmHg. Average serum cholesterol of 15-19 y/o 185 mg/dl, 20-24 y/o 196 mg/dl, 25-34 y/o 210 mg/dl, 35-44 y/o 216 mg/dl, 45-54 y/o 220 mg/dl, 55-64 y/o 217 mg/dl.<br>Females: Average SBP of 15-19 y/o 121 mmHg, 20-24 y/o 116 mmHg, 25-34 y/o 119 mmHg, 35-44 y/o 129 mmHg, 45-54 y/o 141 mmHg, 55-64 y/o 145 mmHg. Average DBP of 15-19 y/o 75 mmHg, 20-24 y/o 75 mmHg, 25-34 y/o 75 mmHg, 35-44 y/o 79 mmHg, 45-54 y/o 86 mmHg, 55-64 y/o 85 mmHg. Average serum cholesterol of 15-19 y/o 198 mg/dl, 20-24 y/o 176 mg/dl, 25-34 y/o 213 mg/dl, 35-44 y/o 223 mg/dl, 45-54 y/o 221 mg/dl, 55-64 y/o 245 mg/dl. | - | - | - | - |
| Prior IA, Stanhope JM, Evans JG, Salmond CE. The Tokelau Island migrant study. *Int J Epidemiol*. Sep 1974;3(3):225-32. doi:10.1093/ije/3.3.225 | Pre-transition: 914 adults 15 years and older living on Atafu, Fakaofo, and Nukunonu. Population was divided into non-migrant and pre-migrant groups.<br>Post-transition: Not evaluated. | A cross sectional study. Data were collected through a series of surveys and a basic physical examination. | - | - | - | Males: Average SBP of 15-19 y/o 115 mmHg, 20-24 y/o 122 mmHg, 25-34 y/o 123 mmHg, 35-44 y/o 126 mmHg, 45-54 y/o 131 mmHg, 55-64 y/o 134 mmHg. Average DBP of 15-19 y/o 66 mmHg, 20-24 y/o 68 mmHg, 25-34 y/o 71 mmHg, 35-44 y/o 77 mmHg, 45-54 y/o 78 mmHg, 55-64 y/o 78 mmHg. Average serum cholesterol of 15-19 y/o 185 mg/100mL, 20-24 y/o 196 mg/100mL, 25-34 y/o 210 mg/100mL, 35-44 y/o 216 mg/100mL, 45-54 y/o 220 mg/100mL, 55-64 y/o 217 mg/100mL<br>Females: Average SBP of 15-19 y/o 121 mmHg, 20-24 y/o 116 mmHg, 25-34 y/o 119 mmHg, 35-44 y/o 129 mmHg, 45-54 y/o 141 mmHg, 55-64 y/o 145 mmHg. Average DBP of 15-19 y/o 75 mmHg, 20-24 y/o 75 mmHg, 25-34 y/o 75 mmHg, 35-44 y/o 79 mmHg, 45-54 y/o 86 mmHg, 55-64 y/o 85 mmHg. Average serum cholesterol of 15-19 y/o 198 mg/100mL, 20-24 y/o 176 mg/100mL, 25-34 y/o 213 mg/100mL, 35-44 y/o 223 mg/100mL, 45-54 y/o 221 mg/100mL, 55-64 y/o 245 mg/100mL | - | - | - | - |
| Salmond CE, Prior IA, Wessen AF. Blood pressure patterns and migration: a 14-year cohort study of adult Tokelauans. *Am J Epidemiol*. Jul 1989;130(1):37-52. doi:10.1093/oxfordjournals.aje.a115321 | Pre-transition: 15-69 y/o in Tokelau in 1968/71, and in 1976. 654 adults were evaluated at all 3 rounds of the study. Subjects were separated into non-migrant and migrant groups.<br>Post-transition: 15-69 living in New Zealand in 1969/70, 1972/74 and 1975/77. 654 people who were present in round I were evaluated again in rounds 2 and 3. | Multiple cross-sectional studies conducted in phases. Data were collected through a series of surveys and a basic physical examination. The data examined in this study is taken from subjects at the beginning and end of the study period. | - | Males: Average BMI 24.8 (non-migrant), 24.1 (migrant)<br>Females: Average BMI 28.8 (non-migrant), 27.8 (migrant) | - | Males: Average SBP 123 mmHg (non-migrant), 121 mmHg (migrant), average DBP 71 mmHg (non-migrant), 70 mmHg (migrant)<br>Females: Average SBP 128 mmHg (non-migrant), 120 mmHg (migrant), average DBP 75 mmHg (non-migrant), 74 mmHg (migrant) | - | Males: Average BMI 26.1 (non-migrant), 28.7 (migrant)<br>Females: Average BMI 27.8 (non-migrant), 31.8 (migrant) | - | Males: Average SBP 128 mmHg (non-migrant), 129 mmHg (migrant), average DBP 74 mmHg (non-migrant), 78 mmHg (migrant)<br>Females: Average SBP 132 mmHg (non-migrant), 129 mmHg (migrant), average DBP 77 mmHg (non-migrant), 77 mmHg (migrant) |
| Stanhope JM, Prior IA. The Tokelau island migrant study: prevalence and incidence of diabetes mellitus. *N Z Med J*. Dec 1980;92(673):417-21. | Pre-transition: All ages living in Tokelau in 1968/71, and in 1976. 1792 people were evaluated.<br>Post-transition: All ages living in New Zealand in 1969/70, 1972/74 and 1975/77. 1567 people were evaluated in Tokelau and 1920 were evaluated in New Zealand. | Multiple cross-sectional studies conducted in phases. Data were collected through a series of surveys and a basic physical examination. | - | - | - | Males: Diabetes prevalence 2.3%, 3.4%<br>Females: Diabetes prevalence 6.1%, 6.5% | - | - | - | Males: Diabetes prevalence 4.9%, 4.8%, 4.4%<br>Females: Diabetes prevalence 3.3%, 6.6%, 10.8% |
| Stanhope JM, Prior IA. The Tokelau Island migrant study: prevalence of various conditions before migration. *Int J Epidemiol*. Sep 1976;5(3):259-66. doi:10.1093/ije/5.3.259 | Pre-transition: 955 adults living in Tokelau in 1968<br>Post-transition: Not evaluated. | Cross sectional study. Data were collected through a questionnaire and basic physical exam. | - | - | Males: 29.3% of the population never smoked.<br>Females: 69.5% of the population never smoked. | Males: Diabetes prevalence 2%, Hypertension prevalence 20%<br>Females: Diabetes prevalence 7.2%, Hypertension prevalence 14.6% | - | - | - | - |
| Stanhope JM, Sampson VM, Prior IA. The Tokelau Island Migrant Study: serum lipid concentration in two environments. *J Chronic Dis*. 1981;34(2-3):45-55. doi:10.1016/0021-9681(81)90050-3 | Pre-transition: All ages living in Tokelau in 1968-1976.<br>Post-transition: All ages living in New Zealand over a period of 1975-1977.1792 people were evaluated. | Multiple cross-sectional surveys. Data were collected through a series of surveys and a basic physical examination. | 1940 kcals, Carbs 35% E, Protein 12% E, Fats 53% E. 45% of fats consumed were saturated, 80% of the saturated fat came from coconuts. | - | - | Males: Average serum cholesterol 4.67 mmol/L<br>Females: Average serum cholesterol 5.14 mmol/L | 2270 kcal, Carbs 44% E, Protein 15% E, Fat: 41% E. Tokelauans now eat more animal products than they did pre-transition. | - | - | Males: Average serum cholesterol 5.09 mmol/L<br>Females: Average serum cholesterol 5.09 mmol/L |
