# Supplementary material for: Dietary Transitions and Health Outcomes in Four Populations – Systematic Review
Source: Front Nutr. 2022 Feb 9;9:748305. doi: 10.3389/fnut.2022.748305 (PMC8892920; doi:10.3389/fnut.2022.748305)
Supplement: Supplementary file 4 [file Data_Sheet_3.PDF]

Tanushimaru	Population Cohort	Methods	Pre-transition				Post-transition			
			Dietary Findings	BMI	Lifestyle	Major Outcomes	Dietary Findings	BMI	Lifestyle	Major Outcomes
Adachi H, Enomoto M, Fukami A, et al. Plasma Renin Activity and Resting Heart Rate in a Population of Community-Dwelling Japanese: The Tanushimaru Study. *Am J Hypertens*. Jul 2015;28(7):894-9. doi:10.1093/ajh/hpu235	Pre transition: N/A<br>Post transition: 1,943 subjects >40 years old living in Tanushimaru in 2009	Prospective cohort study. Data were collected by self report questionnaires comparing different heart rate with different plasma renin activity PRA. Standard physical exam was performed. Results were classified based on heart rate.	-		-	-	-	BMI was categorized based on heart rate: HR<60 (23.4) 60-69 (23.4) 70-79 (23.5) >80 (23.3)	-	No statistically significant associations of all-cause death with PRA. Significant association with high levels of PRA with higher heart rate.<br>SBP: HR <60 (128.2 mmHg) 60-69 (130.8 mmHg) 70-79 (133.4 mmHg) >80 (137.0 mmHg)<br>DBP: HR <60 (78.0 mmHg) 60-69 (79.6 mmHg) 70-79 (82.0 mmHg) >80 (83.6 mmHg)<br>Total Cholesterol: HR<60 (201.7 mg/dl) 60-69 (207.8 mg/dl) 70-79 (209.8 mg/dl) >80 (208.5 mg/dl)
Adachi H, Goetz FC, Jacobs DR, et al. The role of obesity in the association of cardiovascular risk factors and glucose intolerance in small Japanese and North American communities. *Diabetes Res Clin Pract*. Jul 2000;49(1):41-51. doi:10.1016/s0168-8227(00)00133-9	Pre-transition: 210 men and women ages 20-69 from Tanushimaru.<br>Post-transition: 334 men and women >20 in Wadena Minnesota.	Prospective cohort study. Data were collected by comparing the risk factors for cardiovascular disease between Japanese and North American population	-	6.1% (M) and 11.4% (F) of Tanushimaru were overweight and obese.	smoking was controlled for in both studies	Increases in CVD risk factors increased with worsening glucose intolerance, even though BMI remained relatively constant.	-	48.2% (M) and 35.4% (F) of those living in Wadena were overweight and obese.	-	Wadena americans experienced a strong positive correlation between BMI and glucose intolerance.
Adachi H, Hino A. Trends in nutritional intake and serum cholesterol levels over 40 years in Tanushimaru, Japanese men. *J Epidemiol*. May 2005;15(3):85-9. doi:10.2188/jea.15.85	Pre-transition: 628 men aged 40-64 in Tanushimaru in 1958. (Seven Countries Study)<br>Post-transition: 402 men aged 40-64 in Tanushimaru in 1999.	Cohort study. Data were collected in 1958, 1977, 1982, 1989, and 1999. Surveys on dietary patterns, total energy intake, and CVD were distributed.	(1958) 2837 kcal, Carbs 84% E, Protein 11%, Fats 5%<br>Consumed high levels of carbohydrates and low levels of fat	(1958) 21.7	The smoking rate was 68.5%, 68.9%, 62.0% in 1958, 1977 and 1989	Low CHD incidence<br>SBP: 132.9 mmHg DBP: 73.5 mmHg<br>Serum cholesterol: 152.5 mg/dl	(1999) 2202 kcal, Carbs 62% E, Protein 18%, Fats 20%	1999: 23.7	The smoking rate was 57.5%, 45.2% in 1989 and 1999	(1999) Still low CHD incidence - no statistically significant correlation with westernization of diet.<br>SBP 131.6 mmHg DBP 82 mmHg<br>Serum cholesterol: 194.2 mg/dl
Adachi H, Hirai Y, Enomoto M, et al. P1-66 Trends in nutritional intake and serum cholesterol levels over 50 years in Tanushimaru, Japanese men. *Journal of Epidemiology & Community Health*. 2011;65(Suppl 1):A85-A85.	Pre-transition: 628 men aged 40-64 in Tanushimaru in 1958.<br>Post-transition: 329 men aged 40-64 in Tanushimaru in 2009.	Cross-sectional survery (follow-up). Data were collected of dietary patterns/total energy intake and CVD before and after westernization of diet.	(1958) 2837 kcal, Carbs 84% E, Protein 11%, Fats 5%<br>Consumed high levels of carbohydrates and low levels of fat		-	Serum cholesterol: 152.5 mg/dl	(2009) 2289 kcal, Carbs 58% E, Protein 16%, Fats 22%	-	-	Serum cholesterol: 207.7 mg/dl<br>Incidence of CHD remains low.
Hirai Y, Geleijnse JM, Adachi H, Imaizumi T, Kromhout D. Systolic Blood Pressure Predicts Cardiovascular Mortality in a Farming but Not in a Fishing Community–A 40-Year Follow up of the Japanese Cohorts of the Seven Countries Study–. *Circulation Journal*. 2011;75(8):1890-1896.	Pre-transition: 508 men aged 40-64 in Tanushimaru in 1958.<br>Post-transition: Same cohort of men 40 years later.	Cohort study. Mortality was determined from death certificates and hospital records.	-	-	Tanushimaru men had a farming lifestyle, and Ushibuka had a fishing lifestyle			-	-	66.2/10,000 mortality from stroke. 99.8/10,000 mortality from CVD. High SBP carried an increased RR for stroke.
Kimura N, Nakayama Y, Nanbu S, Goda H. Prevalence of plasma lipoprotein abnormalities in the farming village of Tanushimaru and the fishing village of Ushibuka. From the epidemiological aspects. *Jpn Circ J*. Mar 1975;39(3):299-301. doi:10.1253/jcj.39.299	Pre-transition: Seven Countries Study<br>Post-transition: 10 year follow-up of the Seven Countries Study	Prospective cohort study. Data were collected via surveys and results were drawn that compared plasma abnormalities in lipoprotein of a farming village (Tanushimaru) versus a fishing village Ushibuka).	Males: 2271 kcals, Carbs 455g Total Fat 9%E, SFA 3%E, MUFA 3%E, PUFA 3%E, Protein 63g	-	Tanushimaru men had a farming lifestyle, and Ushibuka had a fishing lifestyle	-	-	-	-	Serum cholesterol: 149 mg/dl, high cholestrol was in 4% of the population.<br>Despite different eating habits and lifestyle both populations had similar low frequency of lipoprotein abnormalities and low risk of CHD related mortality
Kimura N, Toshima H, Nakayama Y, Takayama K, Tashiro H, Takagi M. Fifteen-year follow up population survey on stroke: a multivariate analysis of the risk of stroke in farmers of Tanushimaru and Fisherman of Ushibuka. In: Yamori Y, Lovenberg W, Freis ED, eds. *Prophylactic Approach to Hypertensive Diseases*. Raven Press; 1979.	Pre-transition: Seven Countries Study<br>Post-transition:15 year follow-up of the Seven Countries Study	Prospective cohort study. Data were collected via surveys and results were drawn that compared plasma abnormalities in lipoprotein of a farming village (Tanushimaru) versus a fishing village Ushibuka).	Males: 2271 kcals, Carbs 455g Total Fat 9%E, SFA 3%E, MUFA 3%E, PUFA 3%E, Protein 63g	-	Tanushimaru men had a farming lifestyle, and Ushibuka had a fishing lifestyle	9.4% of men had systolic HTN, 8% of men had diastolic HTN	-	-	-	Serum cholesteol at 10th year, 144-155 mg/dl
Kumagae S, Adachi H, Jacobs DR, et al. High level of plasma endothelin-1 predicts development of hypertension in normotensive subjects. *Am J Hypertens*. Oct 2010;23(10):1103-7. doi:10.1038/ajh.2010.125	Pre-transition: 1,492 persons over 40 in Japanesse cohort of the Seven Countries Study<br>Post-transition: 1,261 subjects were re-examined 7 years later.	Cohort study (Follow up). Data were collected from the Seven Countries Study examining blood pressure, BMI, and blood chemistry to compare ET-1 levels in men and women who were hypertensive or non-hypertensive (control)	-	Data was arranged in quartiles of endothelin. Average BMI Q1: 23.0, Q2: 22.9, Q3: 22.6, Q4: 22.0	Smoking (%yes): Q1 30%, Q2 39%, Q3 31%, Q4 48%<br>Alcohol (%yes): Q1 41%, Q2 48%, Q3 37%, Q4 38%	SBP (mmHg): Q1 120, Q2 119, Q3 120, Q4 121<br>DBP (mmHg): Q1 73, Q2 73, Q3 73, Q4 73<br>Total cholestrol (mg/dl): Q1 198, Q2 195, Q3 204, Q4 203	-	-	-	Of the 814 normotensive (baseline) participants who were remeasured during the followup, 222 developed hypertension. Authors conclude that a higher ET-1 does predict probability of developing hypertension.
Menotti A, Keys A, Kromhout D, et al. Inter-cohort differences in coronary heart disease mortality in the 25-year follow-up of the seven countries study. *Eur J Epidemiol*. Sep 1993;9(5):527-36. doi:10.1007/BF00209531	Pre-transition: Seven Countries Study<br>Post-transition: 25 year follow up of Seven Countries Study.	Cohort study (Follow up). Data were collected comparing differences in CHD mortality	-	-	-	Age adjusted death rates from CHD in Tanushimaru men: 45/1,000. Baseline cholesterol 167.9 mg/dl.	-	-	-	Cholesterol decreased about 11mg/dl after 10 years.
Menotti A, Kromhout D, Puddu PE, et al. Baseline fatty acids, food groups, a diet score and 50-year all-cause mortality rates. An ecological analysis of the Seven Countries Study. *Ann Med*. Dec 2017;49(8):718-727. doi:10.1080/07853890.2017.1372622	Pre-transition: Seven Countries Study<br>Post-transition: 50 year follow-up of Seven Countries Study.	Dietary survey. Data were collected on the 7 countries cohort measuring potential dietary correlations with 50-year all cause mortality rates.	2243 kcal, SFA 4%E, MUFA 4%E, PUFA 4.8%E	-	-	<1% obestiy was observed in Japanese cohorts.<br>1960s: the average population intake of saturated (S) and trans (T) fatty acids and hard fats was high in the northern European cohorts while monounsaturated (M), polyunsaturated (P) fatty acids and vegetable oils were high in the Mediterranean areas and total fat was low in Japan. there was correlation between ecological factors, and all cause mortality with monounsaturated over saturated fats. the significance strengthened when controlling for socioeconomic factors	-	-	-	50 year all cause mortality rate: 35.7/1,000
Nakamura S, Adachi H, Enomoto M, et al. Trends in coronary risk factors and electrocardiogram findings from 1977 to 2009 with 10-year mortality in Japanese elderly males - The Tanushimaru Study. *J Cardiol*. Oct 2017;70(4):353-358. doi:10.1016/j.jjcc.2016.12.004	Pre-transition: 231 men 65+ from the Seven Countries Study living in Tanushimaru in 1977.<br>Post-transition: 445 men 65+ living in Tanushimaru in 2009.	Cohort study. Data were collected investigating the trends in coronary risk factors and main ECG findings in 1977, 1989, 1999, and 2009	-	(1977) 20.8	(1977) Smokers 56.7%<br>Alcohol consumption 53%	(1977) SBP: 139 DBP: 75.9<br>Serum cholesterol: 155.8 mg/dl	-	(2009) 23.6	(2009) Smokers 16.8%<br>Alcohol consumption 71.1%	Age, smoking habits, heart rate, and systolic blood pressure were associated with mortality in 1999–2009.<br>SBP: 137.9 DBP: 82<br>Total cholesterol: 192. 5 mg/dl
Takagi M. Serum uric acid as a risk factor for stroke in a fishing village of rural southern Japan. *Jpn Circ J*. Feb 1982;46(2):131-6. doi:10.1253/jcj.46.131	Pre-transition: 314 men (aged 50-79) in the fishing village of Ushibuka.<br>Post-transition: N/A	Multivariate analysis. Data were collected examining health outcomes in Ushibuka subjects over an 8-year period (1970-1978)	-	-	Active fishing village.	30 men experienced stroke during the 8-year period. The mean value of age, systolic blood pressure and serum uric acid were significantly higher in men who experienced stroke, while serum cholesterol, triglyceride, alpha-lipoprotein fraction and albumin were not seen as statistically significant.	-	-	-	-
Toshima H, Koga Y, Menotti A, et al. The seven countries study in Japan. Twenty-five-year experience in cardiovascular and all-causes deaths. *Jpn Heart J*. Mar 1995;36(2):179-89. doi:10.1536/ihj.36.179	Pre-transition: 508 men aged 40-64 in Tanushimaru in 1958.<br>Post-transition: 10 year follow up and prospective analysis of death records at 25 years.	Cohort study. Data were collected comparing the role certain risk factors in each population played in CVD and all cause mortality	-	(1958, 1960) 21.8	(1958, 1960)<br>Prevalence smokers 71%	(1958, 1960) SBP: 167.9, DBP: 73.6, Serum cholesterol: 167.9 mg/dl	-	-	-	Death rates: CHD 46/1,000, strokes 84/1,000. The 25-year death rate for all causes was 30% higher in Ushibuka than in Tanushimaru ($p < 0.001$) and higher also from specific causes except for violence. Baseline differences in the levels of age, blood pressure, serum cholesterol, smoking habits, body mass index and heart rate explained only 19% of the difference in all-cause mortality between the two towns.
Watanabe R, Hanamori K, Kadoya H, Nishimuta M, Miyazaki H. Nutritional intakes in community-dwelling older Japanese adults: high intakes of energy and protein based on high consumption of fish, vegetables and fruits provide sufficient micronutrients. *J Nutr Sci Vitaminol (Tokyo)*. Jun 2004;50(3):184-95. doi:10.3177/jnsv.50.184	Pre-transition: N/A<br>Post-transition: 31 M and 36 W 70+ in Nigata City, Japan	Survey study. Data were collected assessing dietary intake of macronutrients. Nutrient intake was calculated based on the Standard Tables of Food Composition in Japan	-	-	-	-	2,240 kcal, Carbs 59.5%E, Fiber 24.5 g, Protein 16.2%E g, Fat 22.2%E, SFA 14.7 g, MUFA 18 g, PUFA 13.4 g	M: 21.5 F: 23.6	-	From these results, it is evident that age is not an important determinant of dietary intake among apparently healthy elderly Japanese people aged 74 years. In addition, the high intake of energy and protein in the Japanese dietary pattern, based upon high consumption of fish and/or shellfish, vegetables, and fruits, provide sufficient minerals and vitamins.
