# Supplementary material for: Dietary Transitions and Health Outcomes in Four Populations – Systematic Review
Source: Front Nutr. 2022 Feb 9;9:748305. doi: 10.3389/fnut.2022.748305 (PMC8892920; doi:10.3389/fnut.2022.748305)
Supplement: Supplementary file 5 [file Data_Sheet_4.PDF]

YEMENITES	Population Cohort	Methods	Pre-transition				Post-transition			
			Dietary Findings	BMI	Lifestyle	Major Outcomes	Dietary Findings	BMI	Lifestyle	Major Outcomes
Agmon J, Schadel M, Toor M, Rosenzweig B. Diet, fatty acids, and atherosclerosis. A study of aged Yemenite and European immigrants to Israel. Geriatrics. Jun 1966;21(6):159-64	Pre-transition (Yemenite Diet): 220 70+ year old Yemenites living in Israel (1958-1962) for 9-12 years<br>Post-transition (European Diet): 260 70+ year old Europeans living in Israel (1958-1962) for 9-12 years	Observational study. Data were collected by clinical examination (12-lead EKG, venous draw for serum cholesterol) and dietary surveys	2390 total kcal, 381g carbs, 31g protein (animal), 52g protein (vegetable), 19g fat (animal), 40g fat (vegetable) and 22.2% of calories from fats	-	-	Males: 0% MI, 1.1% cases have coronary insufficiency, 5.5% rhythm disturbances, 6.9% conduction disturbances<br>Females: 0% MI, 2.7% cases have coronary insufficiency, 2.7% rhythm disturbances, 2.7% conduction disturbances	2450 total kcal, 351g carbs, 58g protein (animal), 41g protein (vegetable), 30g fat (animal), 42g fat (vegetable) and 26.4% of calories from fats	-	-	Males: 7.7% MI, 5.8% cases have coronary insufficiency, 13.5% rhythm disturbances, 7.7% conduction disturbances<br>Females: 4.6% MI, 5.3% cases have coronary insufficiency, 10.7% rhythm disturbances, 5.3% conduction disturbances
Bavly S. V. Food intake of Yemenite and Kurdish Jews in Israel. Philos Trans R Soc Lond B Biol Sci . Oct 1973;266(876):121-6. doi:10.1098/rstb.1973.0041	Pre-transition (Older Diet): 18-35 y/o Yemenites in 1959 assessed in a prior study<br>Post-transition (Newer Diet): 112 18-35 y/o Yemenite subjects in 1968-1969	Observational study. Data were collected by dietary surveys.	Males: 2850 total kcal, fat 26%E	-	-	-	Males: 3210 total kcal, 466g carbs, 106g protein (total), 40g protein (animal), 104g fat or 29%E<br>Females: 2270 total kcal, 324g carbs, 73g protein (total), 29g protein (animal), 82g fat, Overall: 398g carbs, 93g fat or 31%E<br>Consumed higher levels of meat and fish and lower levels of bread	-	-	-
Brunner D, Meshulam N, Altman S, Bearman JE, Loebl K, Wendkos ME. Physiologic and anthropometric parameters related to coronary risk factors in Yemenite Jews living different time spans in Israel. J Chronic Dis. Aug 1971;24(6):383-92. doi:10.1016/0021-9681(71)90138-x	Pre-transition (Late Arrivals, Yemenite Diet): 126 30-64 y/o (average age was 44/2 +/- 8.2 y/o) male Yemenites who moved to Israel in 1949-1950 Post-transition (Early Arrivals, Israeli Diet): 188 (average age was 42.4 +/- 9.1 y/o) male Yemenites who lived in Israel for at least 30 years	Observational study. Data were collected by clinical examination (anthropometric measures, blood pressures, serum lipoprotein levels, 12-lead EKG).	-	-	Males: physical workers who work on farms.	Males: Average SBP: 116.4 +/- 15.1 mmHg, average DBP: 73.0 +/- 10.0 mmHg, serum cholesterol: 195.0 +/- 39.5 mg% (mg/dL), beta cholesterol: 77.8 +/- 6.4 mg%	-	-	Males: Sedentary workers with moderate amount of physical effort.	Males: SBP 124.4 +/- 16.9 mmHg, DBP 76.1 +/- 9.8 mmHg, serum cholesterol: 208.3 +/- 42.3 mg% (mg/dL), beta cholesterol: 78.6 +/- 6.2 mg% (mg/dL)
Cohen AM. Prevalence of diabetes among different ethnic Jewish groups in Israel. Metabolism. Jan 1961;10:50-8.	Pre-transition (Newcomers, Yemenite Diet): 2463 males and 2443 females both 30-60+ y/o only who immigrated to Israel in the last 10 years<br>Post-transition (Old settlers, Newer Diet): 325 males and 426 females both 3-60+ y/o only who immigrated to Israel over 25 years ago or born in Israel	Observational study. Data were collected from postprandial urine tested immediately by glucose reductase; in cases with positive urine test, a blood sugar and glucose tolerance test was performed.	-	-	-	Males: 0.12% diabetic<br>Females: 0% diabetic<br>All: 0.06%	-	-	-	Males: 4.6% diabetic<br>Females: 1.9% diabetic<br>All: 2.9%
Cohen, AM. Fats and carbohydrates as factors in atherosclerosis and diabetes in Yemenite Jews. Am Heart J. Mar 1963;65:291-3. doi:10.1016/0002-8703(63)90001-2	Pre-transition: 20 "new immigrant" families<br>Post-transition: 20 settled families who lived in Israel for over 24 years	Editorial on past studies. Data were summarized from over observational studies.	Fats were mainly or solely of animal origin and vegetable oil was very rarely used.<br>Carbs consumed were solely or mainly starches with no sugar.	-	-	-	Consumed similar total amounts of animal fat, together with margarine (40-50g daily) and 40 g of vegetable oil. Sucrose accounted for 25-30% of total carbs.	-	-	-
Cohen AM. Environment and prevalence of diabetes in Israel. Tohoku J Exp Med . Dec 1983;141 Suppl:745-54. doi:10.1620/tjem.141.suppl_745	Pre-transition (new immigrant cohort from Cohen 1961 study): 2463 males and 2443 females both 30-60+ y/o only who immigrated to Israel in the from 1950-1960<br>Post-transition (same cohort but 20 years later): 1084 subjects 30+ y/o living in Israel in 1980	Observational study. Data were collected from oral glucose tolerance tests and blood glucose tests.	-	-	-	Males: 0.12% diabetic<br>Females: 0% diabetic<br>All: 0.06%	-	-	-	Males: 8.9% chemical diabetes, 2.7% overt diabetes, 9.2% borderline diabetic<br>Females: 5.5% chemical diabetes, 3.0% overt diabetes, 11.5% borderline diabetic<br>All: 7.0% chemical diabetes, 2.9% overt diabetes, 10.5% borderline diabetic
Cohen AM. Effect of change in environment on the prevalence of diabetes among Yemenite and Kurdish communities. Isr Med J. 1960 May- Jun 1960;19:137-42.	Pre-transition (Newcomers, Yemenite Diet): 2463 males and 2443 females both 30-60+ y/o only who immigrated to Israel in the last 10 years<br>Post-transition (Old settlers, Newer Diet): 325 males and 426 females both 30-60+ y/o only who immigrated to Israel over 25 years ago or born in Israel	Observational study. Data were collected from a previous study (Prevalence of diabetes among different ethnic Jewish groups in Israel, Cohen 1961).	-	-	-	Males: 0.12% diabetic<br>Females: 0% diabetic<br>All: 0.06%	-	-	-	Males: 4.6% diabetic<br>Females: 1.9% diabetic<br>All: 2.9%
Cohen AM, Bavly S, Poznanski R. Change of diet of Yemenite Jews in relation to diabetes and ischemic heart-disease. Lancet. Dec 1961;2(7217):1399-401. doi:10.1016/s0140-6736(61)91215-6	Pre-transition: 108 Yemenites in Israel who immigrated within the last 10 years 15+ y/o; asked about diet while in Yemen (diet data), 64 additional adults from another survey were then further asked about diet while in Israel; 210 additional new immigrants (100 Males and 110 Females) 30+ y/o surveyed for weight and height Post-transition: 112 Yemenites in Israel who lived in Israel from more than 25 years 15+ y/o; asked about current diet in Israel (diet data); 235 additional old settlers (110 Males and 125 Females) 30+ y/o surveyed for weight and height	Observational study. Data were collected from quantitative surveys.	Diet back in Yemen recalled by new immigrants: 2237 +/- 98 total kcal, 343 +/- 15g (58 %E) carbs, 26g mono- and disaccharides, 6.6 +/- 1g sucrose, 80 +/- 3.6g protein (total) with 37% of it is animal, 43 +/- 5g fat (animal), 43 +/- 5g fat (animal + margarine), 14 +/- 2g (oil, 25% of total fat)<br>Diet in Israel by new immigrants (<10 years in Israel): 2468 +/- 120 total kcal, 373 +/- 30g (56 %E) carbs, 76g mono- and disaccharides, 51 +/- 3g sucrose, 78 +/- 4g protein (total) with 49% of it is animal, 43 +/- 8g fat (animal), 49 +/- 12g fat (animal + margarine), 21 +/- 2g (oil, 33% of total fat)	Calculated by JD from avg. height/weight<br>Males: Avg. BMI: 21, avg. weight: 56.5<br>+/- 9.1 kg, avg. height: 162.2 +/- 6.5 cm<br>Females: Avg. BMI: 23.6, avg. weight: 51.3 +/- 9.1 kg, avg. height: 147.6 +/-<br>6.9 cm	-	-	Old settlers: 2559 +/- 118 total kcal, 377 +/- 21g (59 %E) Carbs, 154g mono- and disaccharides, 63 +/- 6g sucrose, 86 +/- 4.4g protein (total) with 46% of it is animal, 42 +/- 7g fat (animal), 51 +/- 3g fat (animal + margarine), 30 +/- 3g (oil, 37% of total fat)	Calculated by JD from avg. height/weight<br>Males: Avg. BMI: 25, avg. weight:<br>64.9 +/- 13.2 kg, avg. height: 160.3 +/- 7.6cm<br>Females: Avg. BMI: 26, avg. weight: 59.5 +/- 9.8 kg, avg. height: 147.6 +/- 150.3 +/- 7.3 cm	-	-
Cohen AM, Fidel J, Cohen B, y/odfat Y, Eisenberg S. Late-onset diabetes in Israel. Isr J Med Sci. Dec 1979;15(12):1003-8	Pre-transition: N/A data already mentioned in previous studies<br>Post-transition: 475 Yemenites from previous survey (299 Males and 176 Females) who immigrated to Israel in 1949-50 and 264 Yemenites were examined in 1968-70 and 211 Yemenites examined in 1975-77 (18-25 years in Israel)	Observational study. Data were collected from oral glucose tolerance tests and blood glucose tests.	-	-	-	-	-	-	-	Males: 14.0% diabetic among all males, 5.0% among 30-44y/o, 12.9% among 45-54y/o, 24.8% among 55+ y/o<br>Females: 10.8% diabetic among all females, 2.6% among 30-44y/o, 17.7% among 45-54 y/o,<br>27.7% among 55+ y/o<br>All: 12.6% diabetic
Cohen AM, Marom L. Diabetes and accompanying obesity, hypertension and ECG abnormalities in Yemenite Jews 40 years after immigration to Israel. Diabetes Res.<br>1993;23(2):65-74.	Pre-transition: N/A<br>Post-transition: 839 Yemenites (490 Males and<br>449 Females) surveyed in 1988-89 (40 years in Israel) who immigrated to Israel in 1948-49	Observational study. Data were collected from postprandial blood glucose test, oral glucose tolerance test, and whole blood glucose from capillary blood draws, BMI's, ECG, and blood pressures also measured	-	-	-	-	-	Males: 12.8% overweight (BMI<br>>/= 27)<br>Females: 6.6% overweight (BMI<br>>/= 25)<br>All: 9.5% overweight	-	Males: 6.2% diabetic, 29.2% with HTN (SBP > 155 mmHg and/or DBP > 95 mmHg)<br>Females: 3.1% diabetic, 35.7% with HTN (SBP > 155 mmHg and/or DBP > 95 mmHg)<br>All: 4.6% diabetic, 31.6% with HTN (SBP > 155 mmHg and/or DBP > 95 mmHg)
Edholm OG, Humphrey S, Lourie JA, Tredre BE, Brotherhood J. VI. Energy expenditure and climatic exposure of Yemenite and Kurdish Jews in Israel. Philos Trans R Soc Lond B Biol Sci . Oct 1973;266(876):127-40. doi:10.1098/rstb.1973.0042	Pre-transition: N/A<br>Post-transition: 75 Yemenites between 20-30 y/o who immigrated to Israel in 1949-1950, data measured in 1968-1969 (~20 years in Israel)	Observational study. Data on daily energy expenditure were collected from time activity survey and measurements of oxygen consumption and heart rates.	-	-	-	-	Males: 2830 total kcal (winter), 3210 total kcal (summer)<br>Females: 2280 total kcal (winter), 2270 total kcal (summer)	-	Males: Spent majority of their time working in the field; daily energy expenditure: 3050 kcal (summer), 3000 kcal (winter); among agricultural workers: 3250 kcal (summer), 3165 kcal (winter)<br>Females: Spent majority of their time doing housework and helping out in the field ; daily energy expenditure: 2280 kcal(summer), 2400 kcal (winter)	-
Glick Z, Shvartz E. Physical working capacity of y/oung men of different ethnic groups in Israel. J Appl Physiol. Jul 1974;37(1):22-6. doi:10.1152/jappl.1974.37.1.22	Pre-transition: N/A<br>Post-transition: 23 Yemenite males between 18- 19 y/o born and raised in Israel	Observational study. Data were collected from anthropometric measures, VO2max, and heart rates.	-	-	-	-	-	Total-Weight: 52.5 kg +/- 6.4 kg; Height: 165 cm +/- 5.2 cm; % Fat: 7.7% +/- 2.8%<br>Students-Weight: 52.6 kg +/- 5.9 kg; Height: 164 cm +/- 8.9 cm; % Fat: 8.2% +/- 2.4%<br>Non-Students: Weight: 52.4 kg +/- 6.3 kg; Height: 165 cm +/- 12.3<br>cm; % Fat: 7.4% +/- 3.0%	Males: Some were students who had physical education 1-2x a week and the non-students worked in various trades, driving, and office work; none of these occupations classified as hard work	-
Kallner G, Groen JJ. Mortality from coronary (arteriosclerotic) heart disease and cerebrovascular accidents among eastern immigrants in Israel. J Chronic Dis. Jan 1968;21(1):25-35. doi:10.1016/0021-9681(68)90083-0	Pre-transition: Yemenites who died in Israel from 1950-1961<br>Post-transition: N/A	Review article. Data were collected on Israeli mortality rates from 1950-1961.	-	-	-	Males: Mortality rate from CVA for 45-64y/o is 48.8 per 100k, for 65+ is 638.5 per 100k; mortality from coronary heart disease for 45-64y/o is 61.7 per 100k, for 65+ is 450.2 per 100k<br>Females: Mortality rate from CVA for 45-64y/o is 61.1 per 100k, for 65+ is 491.4 per 100k; mortality from coronary heart disease for 45-64y/o is 46.2 per 100k, for 65+ is 421.9 per 100k	-	-	-	-
Lehmann EE, Gadoth N, Samueloff S. II. A clinical survey of Yemenite and Kurdish Jews in Israel. Philos Trans R Soc Lond B Biol Sci . Oct 1973;266(876):97-100. doi:10.1098/rstb.1973.0038	Pre-transition: N/A<br>Post-transition: 37 males aged 20-30 and 39 females aged 20-30 in two villages in Israel from March to May 1968	Observational study. Data were collected by clinical examinations (dental health, pulse rates, BP, hematocrit and hemoglobin levels, and level of 'fitness', protein/sugar levels in urine) and surveys on smoking habits, alcohol consumption, and narcotic drugs.	-	-	-	-	-	-	-	Males (Bitha): SBP 127 +/- 10.0 mmHg, DBP 80 +/- 6.2 mmHg; 3/25 'unfit', 22/25 'fit'<br>Males (Peduim): SBP 123 +/- 9.0 mmHg, DBP 77 +/- 9.3 mmHg; 1 'unfit', 11 'fit'<br>Females (Bitha): SBP 127 +/- 16.0 mmHg, DBP 84 +/- 9.0 mmHg; 7 'unfit, including pregnancy',<br>19 'fit'<br>Females (Peduim): SBP 117 +/- 8.7 mmHg, DBP 76 +/- 6.5 mmHg; 5 'unfit, including pregnancy', 8 'fit'

YEMENITES	Population Cohort	Methods	Pre-transition				Post-transition			
			Dietary Findings	BMI	Lifestyle	Major Outcomes	Dietary Findings	BMI	Lifestyle	Major Outcomes
Lubin F, Lusky A, Chetrit A, Dankner R. Lifestyle and ethnicity play a role in all-cause mortality. J Nutr. Apr 2003;133(4):1180-5. doi:10.1093/jn/133.4.1180	Pre-transition: N/A<br>Post-transition: 112 (59 Males and 53 Females) 41-70 y/o people from Yemen/Aden living in Israel in 1969-1983	Observational study. Data were collected from dietary surveys, physical activity questionnaire, weight, blood pressure, smoking habits, and mortality follow-up (1982-2000).	-	-	-	-	3112.5 +/- 1104.5 total kcal/day, 116.9 +/-<br>54.2 g total fat (71.1 +/- 35.2 g unsaturated,<br>43.2 +/- 21.7 g monounsaturated, 27.9 +/-<br>14.3 polyunsaturated, 33.7 +/- 15.7 g saturated); 502.7 +/- 234.3 g cholesterol,<br>34.4 +/- 14.8 g dietary fiber	BMI: 25.0 +/- 3.6	Physical activity: 22.0 +/- 24.0 min/day; 2.5% total E/day alcohol; current smokers: 24.1%, past smokers: 6.2%, non-smokers: 66.1%	SBP: 131.5 +/- 20.6 mmHg. DBP: 84.5 +/- 10.8 mmHg
Lubin F, Lusky A, Chetrit A, Modan M. Differential nutritional habits in distinct ethnic groups in the Israel population. Public Health Rev. 1998;26(1):79-85.	Pre-transition: N/A<br>Post-transition: 116 (62 Males and 54 Females) 40-70 y/o Yemenites living in Israel in the 1990s	Observational study. Data were collected from dietary survey and BMI was also calculated.	-	-	-	-	Males: 3700 +/- 1240 total kcal<br>Females: 2680 +/- 990 total kcal	BMI < 23: 29.5%, BMI 23-26.9:<br>44.6%, BMI 27+: 25.9%	Most Yemenites were physically active (51% were highly active, data not shown) and had lower BMI, a trend that was observed in both sexes.	-
Neufeld HN, Goldbourt U. Coronary heart disease: genetic aspects. Circulation . May 1983;67(5):943-54. doi:10.1161/01.cir.67.5.943	Pre-transition: N/A<br>Post-transition: 280 Yemenite males who have been in Israel for 30 years, 83% emigrated from Yemen in 1948	Systematic Review. Data were collected from several studies assessing incidence of CHD, mortality ratios, and genetic frequencies in various populations including Yemenite Jews.	-	-	-	-	-	-	-	Males (non-CHD cases, n=257) : SBP 131 mm, DBP: 79.6 mm, total cholesterol 198 mg/dL, HDL cholesterol 20.9% of total (n=107)<br>Males (CHD cases, n=23): SBP 149 mm, DBP: 87.5 mm, total cholesterol 197 mg/dL, HDL cholesterol 19.5% of total (n=13)
Samueloff S, Davies CT, Shvartz E. VII. The physical working capacity of Yemenite and Kurdish Jews in Israel. Philos Trans R Soc Lond B Biol Sci . Oct 1973;266(876):141-7. doi:10.1098/rstb.1973.0043	Pre-transition: N/A<br>Post-transition: 36 (22 Males and 14 Females) Yemenites measured in the summer and 25 (18M and 7F) of the same subjects in the winter.<br>Subjects were between 20-30 y/o who immigrated to Israel in 1949-1950, data measured in 1973<br>(~20 years in Israel)	Observational study. Data were collected from exercise with a stationary bike and aerobic power, oxygen intake, ventilation, cardiac frequency, and anthropometric values were measured.	-	-	-	-	-	Males: Weight 63.19 +/- 10.20 kg, Height 163.20 +/- 5.05 cm<br>Females: Weight 51.07 +/- 10.50<br>kg, Height 152.60 +/- 6.79 cm	-	-
Schwartz MJ, Rosensweig B, Toor M, Lewitus Z. Lipid metabolism and arteriosclerotic heart disease in Israelis of Bedouin, Yemenite, and European origin. Am J Cardiol . Aug 1963;12:157-68. doi:10.1016/0002- 9149(63)90305-9	222 patients divided into two groups<br>Pre-transition: Semi-recent Yemenites living in Israel less than 12 years<br>Post-transition: Early Yemenites living in Israel 15- 30 years	Observational study. Data were collected from anthropometric measures, medical and social history, family history, 12-lead EKG, Hb, fasting blood sugar, total serum cholesterol, and an orally administered I131-labeled triolein clearance test.	-	Males: Average weight of 20-34y/o 52.2 kg, 35-59y/o 53.4, 60-90y/o 54.3;<br>average height of 20-34y/o 162.0 cm, 35-59y/o 161.0, 60-90y/o 156.6<br>Females: Average weight of all ages<br>47.6 kg; average height of all ages:<br>156.0 cm	Manual laborers and farmers who lead an active lifestyle; Males: Cigarette smokers 20- 34y/o 6/13, 35-59y/o 3/19, 60-90<br>2/11<br>Females: Cigarette smokers all ages 0/6	Males: Average BP of 20-34y/o 109/72, 35-59y/o 116/78, 60-90y/o 144/74<br>Females: Average BP of all ages 118/72	Consumed higher intake of saturated fatty acids	Males: Average weight of all ages 62.4 kg; average height of all ages: 166.0 cm<br>Females: Average weight of all ages 51.6 kg; average height of all ages: 152.5 cm	Manual laborers and farmers who lead an active lifestyle;<br>Males: cigarette smokers of all ages: 3/9<br>Females: cigarette smokers of all ages: 1/6	Males: Average BP of all ages 125/78 Females: Average BP of all ages 130/79
Schwartz MJ, Schaefer LE. Serum lipids in Americans and Israelis of Yemenite origin. N Y State J Med . May 1 1965;65:1095-100.	Pre-transition: 38 Yemenites (32 Males and 6 Females) who lived in Israel for an average of 12 years or less<br>Post-transition: N/A	Observational study. Data were collected from medical, social, and family history that included occupation, diet, smoking habits, and alcohol consumption; a complete physical examination, a standard 12-lead resting ECG, and levels of blood sugar, total cholesterol, and serum triglyceride after a 12-hour fast.	Males and females consumed a lower caloric and less fatty diet than American Yemenites. Diet includes sunflower seeds, chickpeas, nuts, almonds, vegetables, fruits, yeastless bread, olive and vegetable oils, butter boiled with the seed of fenugreek, and occasionally, meat or chicken.	Males: Average weight 20-34y/o: 115 lbs., 35-65 y/o: 117 lbs.; average<br>height 20-34y/o: 64 in, 35-65 y/o: 63 in<br>Females: Average weight 35-65 y/o: 104 lbs.; average height 35-65 y/o: 61 in	Males: Cigarette smokers (>10 cigarettes/day) 20-34y/o: 46%, 35-65 y/o: 58%; alcohol use in 'moderation'<br>Females: Cigarette smokers (>10 cigarettes/day) 35-65 y/o: 0%; alcohol use in 'moderation'	Males: Average serum cholesterol for 20-34y/o: 195 +/- 22, for 35-65 y/o: 187 +/- 29; average triglyceride for 20-34 y/o: 102 +/- 20, for 35-65 y/o: 99 +/- 30; average blood pressure 25-34y/o: 109/72, 35-65: 116/78<br>Females: Average serum cholesterol for 35-65 y/o: 193 +/- 36; average blood pressure 35-65 y/o: 118/72	-	-	-	-
Toor M, Katchalsky A, Agmon J, Allalouf D. Atherosis and related factors in immigrants to Israel. Circulation . Aug 1960;22:265-79. doi:10.1161/01.cir.22.2.265	Pre-transition: 274 recent Yemenite migrants in Israel<br>Post-transition: 398 early Yemenite migrants in Israel	Longitudinal study. Data was collected with a dietary survey conducted in 2 stages. The first stage was in 1953/54. The second stage was in 1957/58. A basic physical exam including a fasting glucose level was performed.	Males and Females: 1750 Kcal, 66g Protein (12 from animal and 54 from vegetable), 30g Fat (unknown distribution between animal and vegetable)	Males: Average weight (Height Ratio) 35-44y/o: 94.5, 45-54 y/o: 92.4, 55-64<br>y/o: 86.8<br>Females: Average weight (Height Ratio) 35-44 y/o: 93.6, 45-54 y/o: 94.6, 55-64 y/o: 94.9	-	Males: Average total cholesterol of 35-44 y/o is 146.0mg. %, 45-54 y/o is 158mg. %, 55-64 y/o is 158.0<br>Females: Average total cholesterol of 35- 44 y/o is 172.0mg. %, 45-54 y/o is 172mg. %, 55-64 y/o is 190.0.<br>The atherosclerotic mortality rate for the post-transition group (Early Yemenites) aged 45-64 were 4 times the mortality rate than the pre-transition group (Recent Yemenites) aged 45-64y/o.	Males and Females: 2335 Kcal, 91 g Protein (29 from animal and 62 from vegetable), 65g Fat (18 from animal and 47 from vegetable)	Males: Average weight (Height Ratio) 35-44y/o: 100.0, 45-54<br>y/o: 96.0, 55-64 y/o: 93.1;<br>Females: Average weight (Height Ratio) 35-44 y/o: 101.0,<br>45-54 y/o: 108.0, 55-64 y/o: 108.6	-	Males: Average total cholesterol of 35-44 y/o is 188.0mg. %, 45-54 y/o is 197mg. %, 55-64 y/o is 205.0<br>Females: Average total cholesterol of 35-44 y/o is 193.0mg. %, 45-54 y/o is 206mg. %, 55-64 y/o is 224.0.<br>The atherosclerotic mortality rate for the post- transition group (Early Yemenites) aged 45-64 were 4 times the mortality rate than the pre- transition group (Recent Yemenites) aged45- 64y/o.
Weingarten MA, Katzir I, Sprecher E, Kobzantsev S, Zelzer C, Kahan E. Diabetes and ischemic heart disease among Yemenite immigrants in Israel. Isr Med Assoc J. Mar 2000;2(3):207-10.	Pre-transition: N/A<br>Post-transition: 500 charts of 20-44y/o, 250 45-64 y/o, and 350 >64 y/o Yemenites who immigrated between 1949-1951, data collected in 2000	Observational study. Data collected from medical charts and included age, gender, diagnoses, and the most recent serum cholesterol levels.	-	-	-	-	-	-	-	Males: Diabetes prevalence among 25-34y/o: 2%, 35-44y/o: 0.8%, 45-54y/o: 6%, 55-64: 19.2%,<br>65-74y/o: 36.2%, 75+ y/o: 35%<br>Hypertension prevalence among 25-34y/o: 1%%, 35-44y/o: 4.2%, 45-54y/o: 14.9%, 55-64: 23.1%,<br>65-74y/o: 32.8%, 75+ y/o: 23.1%<br>Ischemic heart disease prevalence among 25- 34y/o: 0%, 35-44y/o: 0.8%, 45-54y/o: 1.5%, 55-<br>64: 15.4%, 65-74y/o: 13.8%, 75+ y/o: 21.2%<br>Females: Diabetes prevalence among 25-34y/o: 1.9%, 35-44y/o: 0.9%, 45-54y/o: 10.8%, 55-64:<br>17.2%, 65-74y/o: 30%, 75+ y/o: 20.8%<br>Hypertension prevalence among 25-34y/o: 1%%, 35-44y/o: 0%, 45-54y/o: 3.1%, 55-64: 14.1%, 65-74y/o: 31.4%, 75+ y/o: 27.8%<br>Ischemic heart disease prevalence among 25- 34y/o: 0%, 35-44y/o: 0%, 45-54y/o: 0%, 55-64y/o:<br>3.1%, 65-74y/o: 5.7%, 75+ y/o: 16.7%
